# Tomato R2R3-MYB Proteins SlANT1 and SlAN2: Same Protein Activity, Different Roles

**DOI:** 10.1371/journal.pone.0136365

**Published:** 2015-08-26

**Authors:** Claudia Kiferle, Elio Fantini, Laura Bassolino, Giovanni Povero, Cornelis Spelt, Sara Buti, Giovanni Giuliano, Francesca Quattrocchio, Ronald Koes, Pierdomenico Perata, Silvia Gonzali

**Affiliations:** 1 PlantLab, Institute of Life Sciences, Scuola Superiore Sant'Anna, Pisa, Italy; 2 Italian National Agency for New Technologies, Energy and Sustainable Economic Development, Trisaia Research Center, Rotondella (MT), Italy; 3 Swammerdam Institute of Life Sciences, University of Amsterdam, Graduate School Experimental Plant Sciences, Amsterdam, The Netherlands; 4 Italian National Agency for New Technologies, Energy and Sustainable Economic Development, Casaccia Research Center, Roma, Italy; University of Tsukuba, JAPAN

## Abstract

Anthocyanins are water-soluble polyphenolic compounds with a high nutraceutical value. Despite the fact that cultivated tomato varieties do not accumulate anthocyanins in the fruit, the biosynthetic pathway can be activated in the vegetative organs by several environmental stimuli. Little is known about the molecular mechanisms regulating anthocyanin synthesis in tomato. Here, we carried out a molecular and functional characterization of two genes, *SlAN2* and *SlANT1*, encoding two R2R3-MYB transcription factors. We show that both can induce ectopic anthocyanin synthesis in transgenic tomato lines, including the fruit. However, only SlAN2 acts as a positive regulator of anthocyanin synthesis in vegetative tissues under high light or low temperature conditions.

## Introduction

In plants, anthocyanins accumulate as water-soluble polyphenolic metabolites in the vacuole of many (sub)epidermal cell types, where they exert different functions depending on the tissue specificity. In vegetative tissues anthocyanins act as protective compounds after being synthesized in response to different environmental stimuli (e.g. UV irradiation and/or low temperature) and against pathogens, while in reproductive organs they exert an essential role in attracting pollinators and seed dispersers to assure the reproductive success [1]. Recently, a role for anthocyanins as reducing agents and signaling molecules involved in the modulation of ROS-signaling pathways emerged [2].

Given their physiological importance, the production of anthocyanins must be tightly regulated in plant cells and the regulatory network controlling their biosynthesis has been extensively studied in different species. Several transcription factors (TFs) and other proteins, active not only in vegetative but also in reproductive organs, have been identified (for reviews see [3,4]). In petunia (*Petunia x hybrida*) flowers, for example, different members of the R2R3-MYB family, including *P*. *hybrida* AN2 (PhAN2) [5], two different basic helix-loop-helix (bHLH) proteins, *P*. *hybrida* AN1 (PhAN1) [6] and *P*. *hybrida* JAF13 (PhJAF13) [7], and the WD-repeat (WDR) factor *P*. *hybrida* AN11 (PhAN11) [8], have been associated with the regulation of anthocyanin synthesis. Similarly, MYB, bHLH and WDR factors were found to be responsible of the anthocyanin pigmentation of some fleshy fruits, such as grapevine and apples [4]. In dicots, some of the TFs involved in the production of anthocyanins can individually activate specific early steps of the biosynthetic pathway; others can act in combination to activate transcription of the late biosynthetic structural genes (LBGs) [4]. The analysis of interactions between couples of these factors has brought to propose that a ternary complex constituted by MYB, bHLH and WDR proteins, which is known as MBW complex, is active in this sort of regulation [3], resulting in leaves, floral and fruit pigmentation [4]. Anthocyanins, as well as other plant secondary metabolites, are important phytonutrients and their beneficial effects on health have been demonstrated in a number of intervention studies both in human subjects and animal systems. Anthocyanins have anti-tumor and pro-apoptotic activities as well as anti-oxidative, anti-proliferative, anti-inflammatory, anti-neurodegenerative roles [9,10]. Moreover, anthocyanin-containing plant foods have been reported to prevent type-two diabetes, to reduce low-density lipoprotein (LDL) levels and to improve visual functions by inhibiting myopia and glaucoma [11–14]. For this reason, in the past two decades, there has been a growing interest in the identification of the genetic loci that regulate anthocyanin biosynthesis in major crops as targets for metabolic engineering or breeding programs.

Tomato (*Solanum lycopersicum* L.) is one of the most cultivated vegetable worldwide and its fruits represent a main component of the Mediterranean diet [15]. In many countries, tomato fruits and tomato-based food products are the largest dietary source of lycopene [16], a bioactive red linear carotene which is involved in preventing cardiovascular disease [17] and with chemopreventive effects on prostate cancer cells [18]. Lycopene is the most abundant carotenoid in the ripe fruit, followed by phytoene, phytofluene, β-carotene, ζ-carotene, δ-carotene, lutein, neurosporene and other minor compounds [19], most of which have a bioactive role in human health [20–22]. In addition to carotenoids, tomato fruits contain high amounts of soluble sugars, organic acids, amino acids, and minerals, which, together with hundreds of different volatiles, affect both the taste and the characteristic flavor [23]. Flavonols (mainly quercetin and kaempferol) and flavanones (naringenin), representing the major classes of flavonoids of tomato fruits [24], also contribute to their antioxidant properties. However, the concentration of flavonoids is considered sub-optimal and anthocyanins are generally not present [25–27]. Therefore, this species has been widely used as reference crop for metabolic engineering of the flavonoid pathway and to obtain anthocyanin-enriched tomatoes by using either breeding or transgenic approaches. Constitutive expression of MYB and bHLH regulators of the anthocyanin pathway from other species resulted in the formation of fruits with high levels of anthocyanins [26,28,29]. By a different strategy, interspecific crosses with *Solanum* wild species resulted in purple tomatoes, containing high amount of anthocyanins in the epidermis and the pericarp of the fruits [27,30,31].

Untill now the design of strategies for the breeding or engineering of anthocyanin-rich tomatoes has been limited by the poor knowledge of the regulators of the pathway in this species. In recent years, two members of the R2R3-MYB family have been identified and partially characterized [32–36]. These TFs are encoded by two paralog genes, *S*. *lycopersicum Anthocyanin1* (*SlANT1*) and *S*. *lycopersicum Anthocyanin2* (*SlAN2*), both mapping on chromosome 10 [36] and sharing high similarity with *PhAN2* [32,35]. Constitutive expression of *SlANT1* or *SlAN2*, induced by activation tagging [32] or transgenesis [36,37], caused anthocyanin accumulation in tomato plants, indicating that both MYB TFs activate anthocyanin biosynthetic genes. Interestingly, both *SlANT1* and *SlAN2* are possible candidates for the regulation of the fruit anthocyanin pigmentation in the *Aft* tomato accession [34–36]. Recently, other two MYB encoding genes, *SlMYB7-like* and *SlMYB48-like* have been identified as possible positive regulators of anthocyanin synthesis in tomato and targets of miR858, which acts as a negative regulator of the same pathway [38].

In this study, we present the functional characterization of SlANT1 and SlAN2 in tomato plants. We show that both proteins are able to induce anthocyanin production in the different organs of the plant and demonstrate that the triggering of anthocyanin synthesis by high light or cold in vegetative tissues is mediated by SlAN2, while SlANT1 does not play any role in this mechanism.

## Materials and Methods

### Plant material and growth conditions

The tomato variety Ailsa Craig (AC) (accession LA2838A, Tomato Genetic Resource Center, TGRC, University of California, USA), was used in all experiments, unless otherwise indicated. AC seeds were germinated in rockwool plugs (Grodan, Roermond, the Netherlands) and seedlings were transplanted after two weeks in plastic pots of 10 cm diameter containing a mixture of soil (Hawita Flor, Vechta, Germany) and pumice (70:30, by volume), and placed for other three weeks in a growth chamber (12-h/12-h photoperiod, irradiation intensity 50 μmol photons m^−2^ s^−1^, temperature 24°C, 50% relative humidity). For high light experiments, the plants were then transferred in a 14-h/10-h photoperiod, 28°C temperature, 70% to 80% relative humidity, and light intensity of approx. 300 μmol photons m^−2^ s^−1^. For low temperature experiments, the plants were placed in an incubator set at 15°C, with 14-h/10-h photoperiod, 50 μmol photons m^−2^ s^−1^ and 70% to 80% relative humidity. For RNA and anthocyanin extractions, tissues of apical leaves were sampled at the moment of plants transferring (T-0) and after 2, 4 and 7 days of cold or light treatments, frozen in liquid nitrogen and stored at -80°C until use.

### Phylogenetic analysis

The identification of tomato regulatory anthocyanin genes was carried out using the BLAST search function tool of the Sol Genomics Network (SGN) [39,40] using the genomic sequences of petunia regulatory anthocyanin genes as query. The deduced amino acid sequences of 20 genes encoding R2R3-MYB proteins, 14 genes encoding bHLH factors and 12 genes encoding WDR proteins were aligned using the MUSCLE algorithm in the MEGA 6 package [41]. The results of the phylogenetic analysis was visualized by the Neighbor-Joining method [42] through MEGA 6. The statistical reliability of individual nodes was assessed by bootstrap analysis with 1000 replicates and the evolutionary distances were computed using the p-distance method. For the analysis of R2R3-MYB factors we used the MYB domain of the following proteins: SlANT1 (AAQ55181.1); SlAN2 (FJ705320.1); *S*. *lycopersicum* ANT1like, SlANT1like (ACT366161); *S*. *lycopersicum* AN2like, SlAN2like (ACT366117.1); *Solanum tuberosum* AN1, StAN1 (AAX53089.1); *S*. *tuberosum* AN2, StAN2 (AAX53091.1); PhAN2 (ABO21074.1); *P*. *hybrida* DPL, PhDPL (HQ116169); *P*. *hybrida* PHZ, PhPHZ (HQ116170); *P*. *hybrida* PH4, PhPH4 (BAP28594.1); *P*. *hybrida* ODO1, PhODO1 (Q50EX6.1); *Nicotiana tabacum* AN2, NtAN2 (ACO52470.1); *Antirrhinum majus* ROSEA1, AmROS1 (ABB83826.1); *A*. *majus* ROSEA2, AmROS2 (ABB83827.1); *Arabidopsis thaliana* MYB75, AtMYB75 (AAG42001.1); *A*. *thaliana* MYB113, AtMYB113 (NM_105308); *A*. *thaliana* MYB114, AtMYB114 (NM_105309); *Zea mays* C1, ZmC1 (AAA33482); *Z*. *mays* Pl, ZmPl (AAA19819); *Malus domestica* MYB10, MdMYB10 (ABB84753). The bHLH factors and their GenBank accession numbers are as follows: PhAN1 (AAG25927); PhJAF13 (AAC39455); *A*. *thaliana* GL3, AtGL3 (NP_680372); *A*. *thaliana* EGL3, AtEGL3 (NP_176552); *A*. *thaliana* TT8, AtTT8 (CAC14865); *Z*. *mays* IN1, ZmIN1 (AAB03841); *Z*. *mays* Lc, ZmLc (NP_001105339); *M*. *domestica* bHLH, MdbHLH (ADL36597); *Citrus x sinensis* MYC2, CsMYC2 (ABR68793); *A*. *majus* DELILA, AmDEL (AAA32663); *N*. *tabacum* AN1a, NtAN1a (HQ589208.1); *N*. *tabacum* AN1b, NtAN1b (HQ589209.1); *S*. *lycopersicum* AN1, SlAN1 (this study); *S*. *lycopersicum* JAF13, SlJAF13 (this study). The WDR factors and their GenBank accession numbers are as follows: PhAN11 (U94748.1); *A*. *thaliana* TTG1, AtTTG1 (NM_180739.2); *Z*. *mays* PAC1, ZmPAC1 (AY115485.1); *S*. *lycopersicum* AN11, SlAN11 (this study); *Ipomoea purpurea* WD40, IpWD40 (ABW69689.1); *N*. *tabacum* TTG2, NtTTG2 (ACN87316.1); *S*. *tuberosum* TTG1-like, StTTG1-like (XP_006347612.1); *M*. *domestica* TTG1-like, MdTTG1-like (XP_008343816.1); *Gossypium hirsutum* TTG1, GhTTG1 (AAK19614.1); *G*. *hirsutum* TTG3, GhTTG3 (AAM95645.1); *Fragaria x ananassa* TTG1, FaTTG1 (AFL02466.1); *Prunus persica* TTG1, PpTTG1 (ACQ65867.1).

These sequences were used in a second round of alignment and phylogenetic analysis, performed as described above, including 96 R2R3-MYB, 98 bHLH and 66 WDR amino acid sequences annotated in the tomato genome.

### RNA-Seq in tomato tissues

Data from ILLUMINA RNA-Seq experiment were used to generate an expression heatmap of the genes involved in anthocyanin accumulation. The data are expressed as the average of FPKM (Fragments Per Kilobase of exon model per Million mapped fragments) values obtained from two biological replicates of 7 tissues of *S*. *lycopersicum* cv. Moneymaker (accession LA2706, TGRC): root, stem, leaf, flower and fruit at mature green, breaker and ripe (10 days post breaker) developmental stages. The data were processed with the software Genesis 1.7.6 (Gene Expression Similarity Investigation Suite, [43]), and visualized with a log2 scale to reduce the saturation effect of highly expressed genes.

### Promoter sequence analysis

The 2 kb nucleotide genomic sequences of *SlANT1* and *SlAN2* were obtained from the Sol Genomics Network [39,40]. Cis-acting promoter regulatory elements in *SlANT1* and *SlAN2* promoters were identified through the PlantCARE database [44].

### Cloning of the MYB genes

AC genomic DNA was extracted from a single leaf using the “Wizard Genomic DNA Purification Kit” (Promega, Madison, WI, USA). *SlANT1* and *SlAN2* were amplified by PCR starting from AC genomic DNA using the “Phusion High-Fidelity DNA Polymerase” (New England Biolabs Inc., MA, USA) and the following pairs of primers: CACCATGAACAGTACATCTATGTC (forward) and TTAATCAAGTAGATTCCATAAGTCAA (reverse) for *SlANT1*; CACCATGAATACTCCTATGTGTGC (forward) and TTAATTAAGTAGATTCCATAAGTCAATATC (reverse) for *SlAN2*. The amplified sequences were cloned into pENTR/D-TOPO vector (Life Technologies, Carlsbad, CA, USA) and the entry clones were recombined with different destination vectors, as described below, via “Gateway Recombination Cloning Technology” (Life Technologies).

### Expression analysis by quantitative RT-PCR (qPCR)

Total RNA was extracted from tomato plants using a “Spectrum Plant Total RNA Kit” (Sigma–Aldrich, St Louis, MO, USA). RNA was subjected to DNase treatment using a “TURBO DNA free Kit” (Life Technologies) and then reverse transcribed into cDNA with an “iScript cDNA Synthesis Kit” (Bio-Rad Laboratories, Hercules, CA, USA). qPCR was performed with an ABI Prism 7300 Sequence Detection System (Applied Biosystems, Foster City, CA, USA) using the “iTaq Universal SYBR Green Supermix” (Bio-Rad) and the primers listed in S1 Table. *S*. *lycopersicum elongation factor 1-alpha* (*SlEF1A*) was used as reference gene. The relative quantification of each individual gene expression was performed using the geometric averaging method (geNorm) [45].

### SlAN2 and SlANT1 ectopic expression *in planta*

The 35S:*SlANT1* and 35S:*SlAN2* constructs were produced by recombining the *SlANT1* and *SlAN2* entry clones with the Gateway compatible binary vector pK7WG2 [46] (http://gateway.psb.ugent.be/). Tomato plants ectopically expressing *SlANT1* and *SlAN2* were produced by *Agrobacterium tumefaciens*-mediated transformation [29]. For RNA extraction and qPCR analyses, leaves, petals, anthers, peel and flesh from fruits at the mature green stage [47] isolated from single representative transgenic lines [line T9002 with 35S:*SlANT1* (T1 generation) and line R9009 with 35S:*SlAN2* (T1 generation)] were used. The anthocyanin-rich phenotype of the T9002 and R9009 transgenic lines was inheritable.

### Anthocyanin quantification

Anthocyanin extraction was performed as described by [48] starting from 0.5 mg of apical leaves. The total amount of anthocyanins was expressed as mg petunidin-3-(p-coumaroyl rutinoside)-5-glucoside per gram fresh weight, as described in [28]. Mean values were obtained from three independent replicates.

### Protein localization assays in protoplasts

For localization in protoplasts, the entry clones of *SlANT1* and *SlAN2* were recombined with the Gateway destination vector p2FGW7 [46]. *Arabidopsis* mesophyll protoplasts were isolated from rosette leaves and transfected according to [49]. Fluorescence was imaged with a Nikon Eclipse Ti-5 video-confocal microscope using Endow GFP and DAPI filters.

### Transactivation Assay

Transactivation assay was performed exploiting the *Renilla reniformis* (Rr) and *Photinus pyralis* (Pp) luciferase enzymes. The 35S:*SlANT1* and 35S:*SlAN2* effector constructs were produced by recombining the *SlANT1* and *SlAN2* entry clones with the vector p2GW7 [46]. The *SlDFR* promoter was amplified from AC genomic DNA using the primers pDFR_GWFW (CACCTTAGTGAAAGACCAACGTG) and pDFR_GWRV (TTTCAGAAATGAAAGGTAAAAAAGAGTC), cloned into pENTR/D-TOPO vector and then recombined with the reporter plasmid pPGWL7 containing the PpLuc gene. A RrLuc-overexpressing vector [50] was used to normalize luminescence values detected in protoplasts. Both effector and reporter plasmids were co-transformed in Arabidopsis mesophyll protoplasts, isolated as described above, and the relative levels of luciferase were measured, as described in [50]. Luminescence was measured with a Lumat LB 9507 Tube Luminometer (Berthold Technologies, NY, USA).

### Virus Induced Gene Silencing (VIGS)

TRV-based T-DNA binary vectors pTRV1, pTRV2 and pTRV2/GATEWAY are from [51]. For both *SlAN2* and *SlANT1*, a fragment of the cDNA was amplified using primers designed in order to introduce attB Gateway cloning sites: AN2_attB1: GGGGACAAGTTTGTACAAAAAAGCAGGCTTTGCATTGAAATTGAAGAAG, AN2_attB2: GGGGACCACTTTGTACAAGAAAGCTGGGTTCCATAAGTCAATATCAGTT, ANT1_attB1:GGGGACAAGTTTGTACAAAAAAGCAGGCTAGAAAAATCACCACCATTAAAT, ANT1_attB2:GGGGACCACTTTGTACAAGAAAGCTGGGTTTCCATAAGTCAATTTCAGCA. The fragment was cloned into the pTRV2 using the “Gateway Recombination Cloning Technology” (Life Technologies). *Agrobacterium* cultures were grown as described in [52], and cell concentration in the infiltration media was adjusted to an OD of 0.1. Tomato seedlings (Money Maker variety) were vacuum-infiltrated [53] with a 1:1 (v/v) mixture of two *A*. *tumefaciens* C58C1 strains, containing the pTRV1 and the pTRV2 binary vectors with the silencing fragment (or the empty pTRV2 as control) respectively. Infiltrated seedlings were plenty washed and kept in the dark for at least 12 hours and then grown under stressing conditions to promote anthocyanins accumulation: low temperature (17°C) and limiting soil (e.g. 3 plants in a 80x80x90 mm pot). The RNA was extracted from silenced and non-silenced tissues of three biological replicates and the expression levels of regulatory and target genes were measured as described above.

## Results

### Identification of tomato candidate anthocyanin regulatory genes

**Fig 1:**
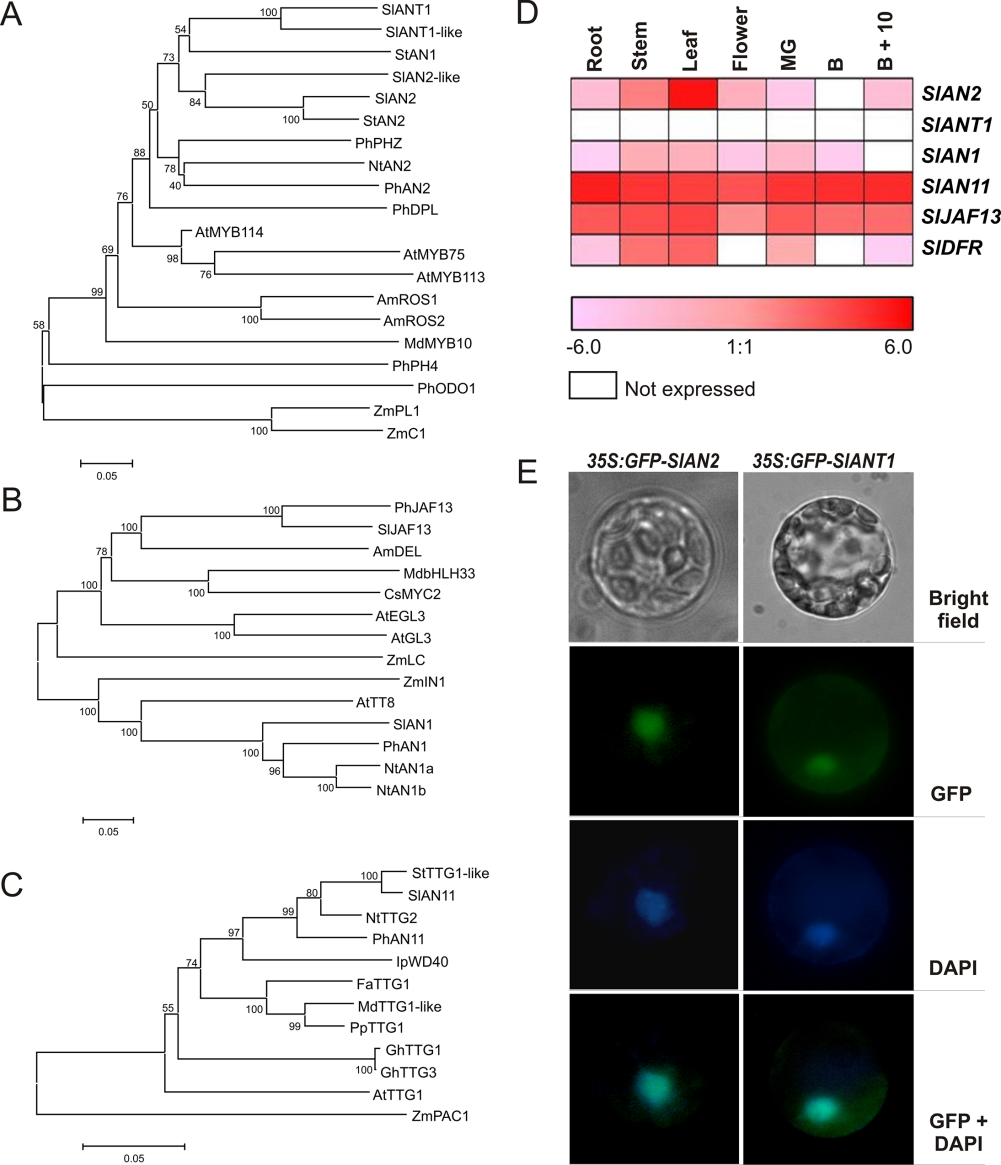
Identification of possible tomato R2R3-MYB, bHLH, and WDR regulators of anthocyanin synthesis. Evolutionary relationships of R2R3-MYB (A), bHLH (B) and WDR (C) proteins involved in anthocyanin pigmentation in different plant species. The evolutionary history was inferred using the Neighbor-Joining method [42]. The optimal tree with the sum of branch length (A = 4.04792561, B = 3.00531532, C = 0.83964559) is shown. The percentage of replicate trees in which the associated taxa clustered together in the bootstrap test (1000 replicates) are shown next to the branches [57]. The tree is drawn to scale, with branch lengths in the same units as those of the evolutionary distances used to infer the phylogenetic tree. The evolutionary distances were computed using the p-distance method [58] and are in the units of the number of amino acid differences per site. The analysis involved 20 (A), 14 (B) and 12 (C) amino acid sequences. All ambiguous positions were removed for each sequence pair. There were a total of 790 (A), 364 (B) and 372 (C) positions in the final dataset. Evolutionary analyses were conducted in MEGA6 [41]. Expression heatmap (Log2 scale of FPKM values) of *SlAN2*, *SlANT1*, *SlAN1*, *SlAN11*, *SlJAF13* and *SlDFR* genes in different tissues of tomato, analyzed by Illumina RNA-Seq (D). MG: Mature Green fruit; B: Breaker fruit; B+10: ripe fruit 10 days after breaker stage. Subcellular localization of GFP-SlANT1 and GFP-SlAN2 fusion proteins in transiently transformed *A*. *thaliana* mesophyll protoplasts (E). Pictures were taken with bright field, green fluorescent protein (GFP) and 4’6-diamidino-2-phenylindole (DAPI) filters.

By analyzing the whole genome of tomato, all the annotated putative MYB proteins were compared with the main MYB regulatory factors involved in anthocyanin synthesis in other species (S1 Fig). Four different tomato MYB proteins, encoded by the genes *Solyc10g086250*, *Solyc10g086260*, *Solyc10g086270*, and *Solyc10g086290*, corresponding, respectively, to SlAN2 [33–35], SlANT1 [32,36], SlANT1like [35,54] and SlAN2like [35], grouped in one clade with MYB proteins of tobacco (*N*. *tabacum*), petunia and potato (*S*. *tuberosum*) involved in anthocyanin synthesis [5,55,56] (S1 Fig, Fig 1A). This clade included members from the *Solanaceae* family and was clearly separated from anthocyanin MYBs from other dicots, such as *A*. *thaliana* or *A*. *majus*, and monocots (S1 Fig, Fig 1A). This analysis confirms that SlANT1 and SlAN2 are indeed tomato MYB TFs involved in anthocyanin synthesis regulation.

To identify possible tomato regulators belonging to the bHLH and WDR families to be included in our analyses, a similar phylogenetic approach was followed. In this way we could identify two distinct bHLH factors, encoded by the genes *Solyc09g065100* and *Solyc08g081140*, which group with the major plant bHLH factors involved in anthocyanin synthesis (S2 Fig). These two proteins show strong homology respectively to PhAN1 and PhJAF13 and were thus named SlAN1 and SlJAF13. The genes *Solyc09g065100* and *Solyc08g081140* likely correspond to the sequences mapped by [33] on chromosomes 9 and 8 of tomato and already named *an1* and *jaf13* for their homology with *PhAN1* and *PhJAF13*, respectively. SlAN1 and SlJAF13 belong to two different clades of bHLH anthocyanin regulatory factors, the first one including *A*. *thaliana* TT8 and *Z*. *mays* IN1, and the second one *A*. *thaliana* GL3 and EGL3, *A*. *majus* DELILA, and *Z*. *mays* LC (S2 Fig, Fig 1B).

Finally, we identified a tomato anthocyanin-related WDR protein, encoded by the gene *Solyc03g097340*, by homology with the petunia PhAN11 (S3 Fig) and we named this protein SlAN11. *Solyc03g097340* likely corresponds to the sequence mapped on chromosome 3 of tomato by [33] and already named *an11* for its homology with *PhAN11*. SlAN11, as expected, groups with other dicot WDR proteins, such as PhAN11 and AtTTG1, while the *Z*. *mays* protein PAC1 is more distantly related (S3 Fig, Fig 1C).

The expression pattern of these tomato MYB, bHLH and WDR genes in plants grown in standard conditions was obtained from RNA-Seq data in 7 different tissues: root, stem, leaf, flower and fruit at mature green, breaker and ripe developmental stages (S2 Table; Fig 1D). The transcript of the LBG *SlDFR*, encoding a key enzyme in the anthocyanin biosynthetic pathway, was included in the analysis. In the heatmap of Fig 1D, *SlAN2* shows the highest levels of expression in leaves, followed by stems. The expression pattern of *SlAN1* and *SlDFR* is similar to *SlAN2; SlAN11* and *SlJAF13* transcripts are expressed in all analyzed organs, whereas *SlANT1* expression was not detected in any tissue. An analysis carried out in a wider dataset of tomato tissues or developmental stages showed that the level of expression of *SlANT1* is indeed always quite low in comparison to those of *SlAN11* and *SlJAF13* (S4 Fig).

The subcellular localization of the GFP fusions of SlANT1 and SlAN2 was assessed by expressing them under the control of the *Cauliflower Mosaic Virus 35S* promoter (35S) in transiently transformed protoplasts of *A*. *thaliana*. Both SlAN2 and SlANT1 localized into the cell nucleus (Fig 1E), consistently with their possible role as TFs.

### Expression profile of the MYB, bHLH, and WDR genes in lines ectopically expressing SlANT1 and SlAN2

**Fig 2:**
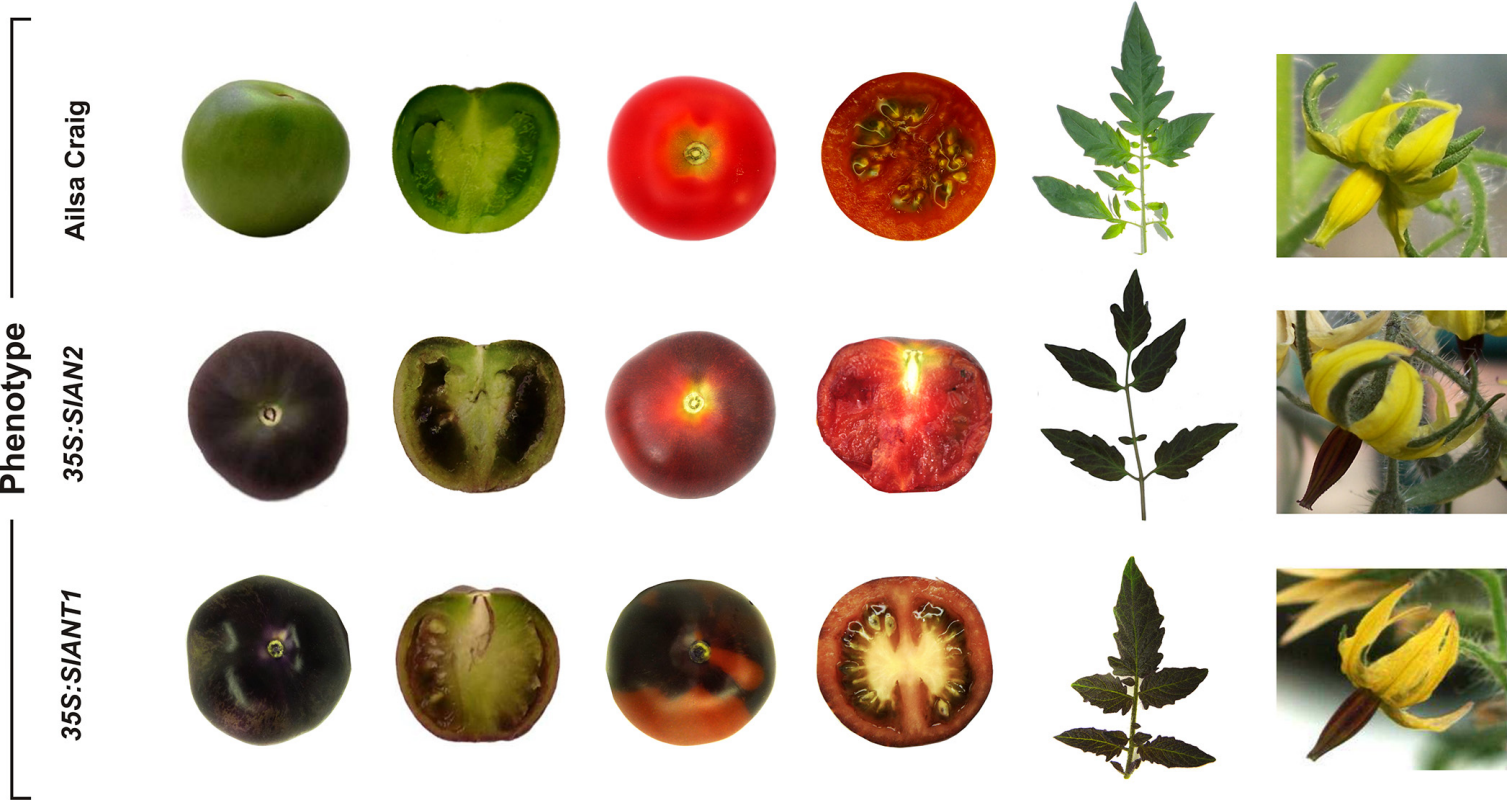
35S:*SlANT1* and 35S:*SlAN2* transgenic tomato lines. Phenotype of 35S:*SlAN2* and 35S:*SlANT1* lines compared to the control non-transformed plants (Ailsa Craig). Details of immature green fruits, section of the immature green fruits, red ripened fruits, section of the red fruits, leaves, and flowers are shown. 35S:*SlANT1* line T9002 and 35S:*SlAN2* line R9009 were chosen for the phenotypic analysis.

To study the effect of the ectopic expression of SlAN2 and SlANT1 *in planta*, we generated tomato lines expressing *SlAN2* or *SlANT1* under the control of the *35S* promoter. Eleven independent transgenic lines for 35S:*SlANT1* and 12 independent lines for 35S:*SlAN2* were produced. All transgenic lines showed enhanced anthocyanin synthesis, resulting in a strong pigmentation of vegetative organs, flowers and fruits (Fig 2). This is in agreement with previous evidences obtained in tomato with *SlANT1* [32,36] and *SlAN2* [37]. Our results indicate that SlAN2 and SlANT1 ectopic expression results in similar phenotypes and indicate that the SlANT1 and SlAN2 proteins are equally able to activate the anthocyanin biosynthetic pathway in tomato. In leaves, anthocyanin accumulated both in leaflets and in leaf rachis; in flowers, pigmentation was mainly visible in the anthers (Fig 2). In both 35S:*SlAN2* and 35S:*SlANT1* plants, anthocyanins accumulated in fruit till the immature green stage resulting in intense pigmentation of the peel and in the surface of the locular cavities in immature green fruits (Fig 2). However, with fruit maturation, anthocyanin pigmentation of the peel progressively reduced in 35S:*SlAN2* lines, while this was not observed in 35S:*ANT1* fruits that remained strongly colored, although not homogeneously (Fig 2). This could be the consequence of prolonged anthocyanin synthesis in the fruits of 35S:*SlANT1* plants, and/or prolonged persistence of the pigments till late ripening. In 35S:*SlAN2* tomatoes instead, pigments present in the peel at the immature green stage, appeared to be diluted during further growth and ripening of the fruits, as if synthesis of anthocyanins arrested. Recently, Meng et al. [59] found that physiological changes in tomato fruit ripening were caused by overexpression of *SlAN2*. In particular, an increased ethylene synthesis as well as a reduction of carotenoid levels, including lycopene, with a consequent orange colour of the fruits at ripening, were found associated to *SlAN2* overexpression. These changes may reflect a possible peculiar role of this TF as a regulator of fruit ripening, in addition to trigger of anthocyanin synthesis.

**Fig 3:**
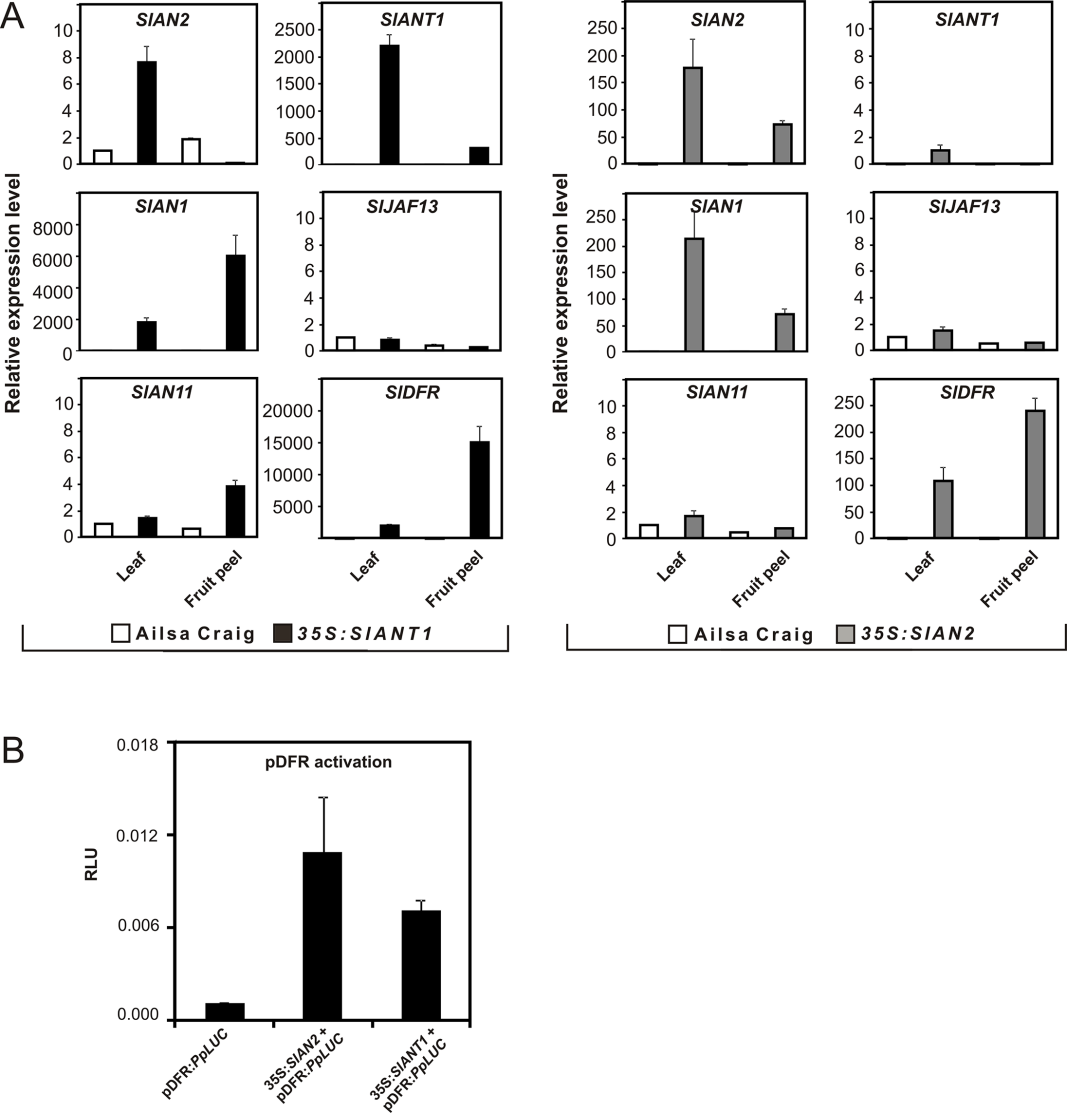
Effect of the overexpression of *SlANT1* and *SlAN2* on other genes of the anthocyanin pathway. Quantitative analysis of transcript levels of *SlAN2*, *SlANT1*, *SlAN1*, *SlAN11*, *SlJAF13* and *SlDFR* in leaves and peel from green fruits of the 35S:*SlANT1* and 35S:*SlAN2* lines in comparison with control Ailsa Craig plants (A). Expression levels are shown as relative units, with the value of AC leaves set to one. A sample composed of two biological replicates was analyzed for each plant tissue and data are means of two technical replicates ± SD. 35S:*SlANT1* line T9002 and 35S:*SlAN2* line R9009 were chosen for the qPCR analysis. Transient transformation experiment in Arabidopsis mesophyll protoplasts showing that both SlAN2 and SlANT1 activated the *SlDFR* promoter (B). Protoplasts were transfected with the reporter plasmid containing the *SlDFR* promoter driving firefly luciferase (PpLuc) gene alone (first histogram) or in combination with the effector plasmid containing either the full length *SlAN2* or *SlANT1* coding sequence (second or third histograms, respectively). A 35S:Renilla-luciferase (RrLuc) plasmid was used as an internal control. Data are expressed as Relative Luc Activity (RLU) (PpLuc/RrLuc) and are means of eight biological replicates ± SE.

The intense anthocyanin pigmentation observed in many organs of the transgenic lines demonstrated that the ectopic expression of a single endogenous R2R3-MYB factor (SlAN2 or SlANT1) was sufficient to activate the anthocyanin biosynthetic pathway. This indicated that the other regulators of the pathway were already expressed where anthocyanin accumulated or that their expression was induced by SlAN2 and SlANT1. To verify these hypotheses we examined by qPCR the expression pattern of the putative regulatory genes in the transgenic lines. Representative plants of single 35S:*SlANT1* and 35S:*SlAN2* lines, characterized by a strong anthocyanin phenotype, were selected for the analysis. In both transgenic lines, the expression of either *SlANT1* or *SlAN2* resulted in strong induction of the bHLH gene *SlAN1* and of the LBG gene *SlDFR* in leaves and fruit peel (Fig 3A). Furthermore, in leaves, small activation effects on *SlAN2* in the *SlANT1* transgenic line, and on *SlANT1* in the *SlAN2* transgenic line were also observed (Fig 3A). On the contrary, the expression levels of the other bHLH gene, *SlJAF13*, and of the WDR gene *SlAN11* appeared to be similar in wild type and transgenic plants (Fig 3A).

A transactivation assay carried out in *A*. *thaliana* protoplasts showed that both SlANT1 and SlAN2 can activate the promoter of *SlDFR* (Fig 3B).

Together, these results indicate that the MYB factors SlAN2 and SlANT1 can induce the transcription of *SlDFR* by activating transcription of the bHLH gene *SlAN1*. This is in agreement with previous observations that MYB proteins in other species regulate the transcription of their bHLH partners and subsequently form together with the bHLH protein and the WDR one (which is expressed in all plant parts, [8]) a MBW complex that activates the structural genes [6,60]

### SlAN2 is involved in the up-regulation of the anthocyanin pathway upon light and cold stress

**Fig 4:**
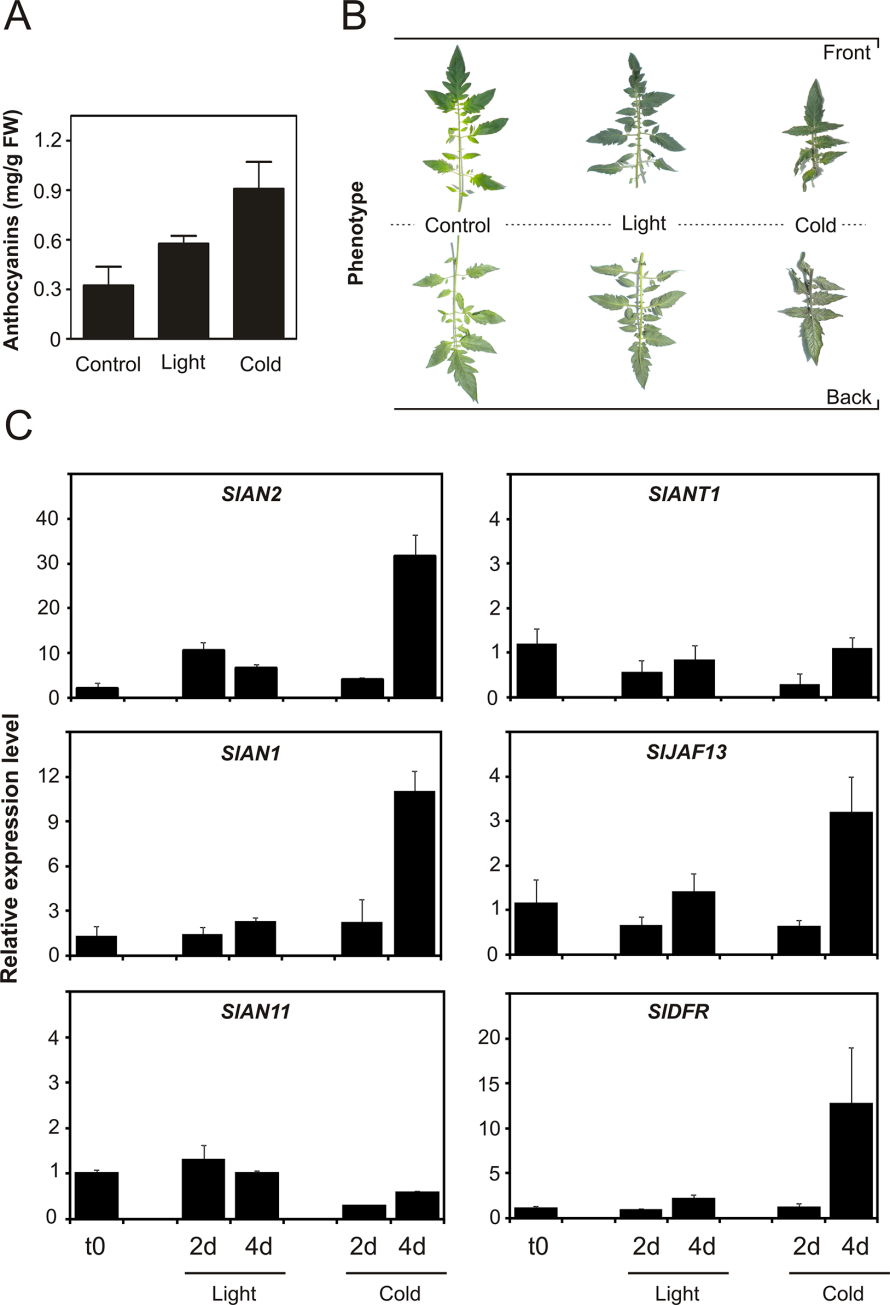
Induction of anthocyanin synthesis in tomato plants under high light and low temperatures conditions. Anthocyanin content in leaves from Ailsa Craig plants treated for 7 days with high light (approx. 300 μmol photons m^−2^ s^−1^) or low temperature (15°C) compared to untreated control plants (A) and phenotypes of the same leaves (B). Quantitative analysis of transcript levels of selected anthocyanin genes in vegetative tissues subjected to 2 and 4 days of high light or low temperature treatments (C). Expression levels are shown as relative units, with the value of one of the biological replicates of control untreated samples set to one. Data are means of three biological replicates ± SD.

To investigate whether *SlANT1* and *SlAN2* are modulated by environmental factors, we analyzed their expression in vegetative tissues of 4-week-old tomato plants that were exposed for 7 days to high light or low temperature conditions. These factors are known to be major triggers of anthocyanin synthesis and accumulation in plant green parts [1]. Anthocyanin synthesis was activated during the exposure to high light or low temperature, as confirmed by both the analysis of the anthocyanin content of the leaves at the end of the treatments (Fig 4A) and the phenotype of the leaves themselves (Fig 4B). qPCR analysis, carried out after 2d and 4d of light or low temperature, showed the induction of *SlAN2* following both treatments (Fig 4C). Moreover, a slight induction of *SlAN1* and *SlDFR* was observed as a consequence of the light treatment, particularly after 4d (Fig 4C). More pronounced was the activation of the same genes, as well as of *SlJAF13*, during the low temperature treatment (Fig 4C). *SlAN11* expression was not particularly affected by high light or cold (Fig 4C), suggesting that there was not a direct correlation with the activation of the pathway and probably that basal *SlAN11* expression levels were sufficient to induce anthocyanin synthesis. *SlANT1* is expressed at very low levels at standard growth conditions, as seen in Figs 1D and 3A. Moreover no activation was detected upon light or cold induction (Fig 4C). This indicates that SlANT1 does not contribute to the activation of the anthocyanin biosynthetic pathway neither under standard growth condition, nor upon stress conditions by high light and low temperature.

The analysis of a 2 kb nucleotide genomic sequence upstream of *SlAN2* indicated the presence of several cis-acting elements involved in light responsiveness, such as GATA-motifs, Box 4 and I-box elements and many others (S5 Fig). Specific cold responsive elements were not found. However, cold-responsive genes can also be regulated through cis-acting abscisic acid response elements (ABREs) [61] and some ABREs were identified in the promoter region of *SlAN2* (S5 Fig). Furthermore, jasmonate responsive elements and defense and stress-responsive elements, compatible with other developmental and environmental triggers of anthocyanin synthesis [1,62], were found (S5 Fig). The presence of all these cis-acting elements, particularly the high number of light responsive elements (LREs), was a further indication of the involvement of SlAN2 in the regulation of anthocyanin synthesis, particularly when induced by light. However, the analysis carried out on the promoter sequence of *SlANT1* highlighted the presence of similar categories of regulatory elements (LREs, ABREs, defense, stress and jasmonate responsive elements) (S6 Fig). Moreover, additional and specific cis-acting regulatory elements, potentially involved in other hormonal and developmental mechanisms controlling anthocyanin synthesis, such as gibberellin and drought [1,62], were found (S6 Fig). It is thus possible that *SlANT1* transcription is induced in conditions different from those controlling *SlAN2*, even if we cannot exclude that light conditions different from the ones tested in our experimental set-up (for example higher) could also activate *SlANT1* transcription, due to the high number of LREs identified in its promoter.

### SlAN2 plays a major role in the activation of anthocyanin biosynthesis

**Fig 5:**
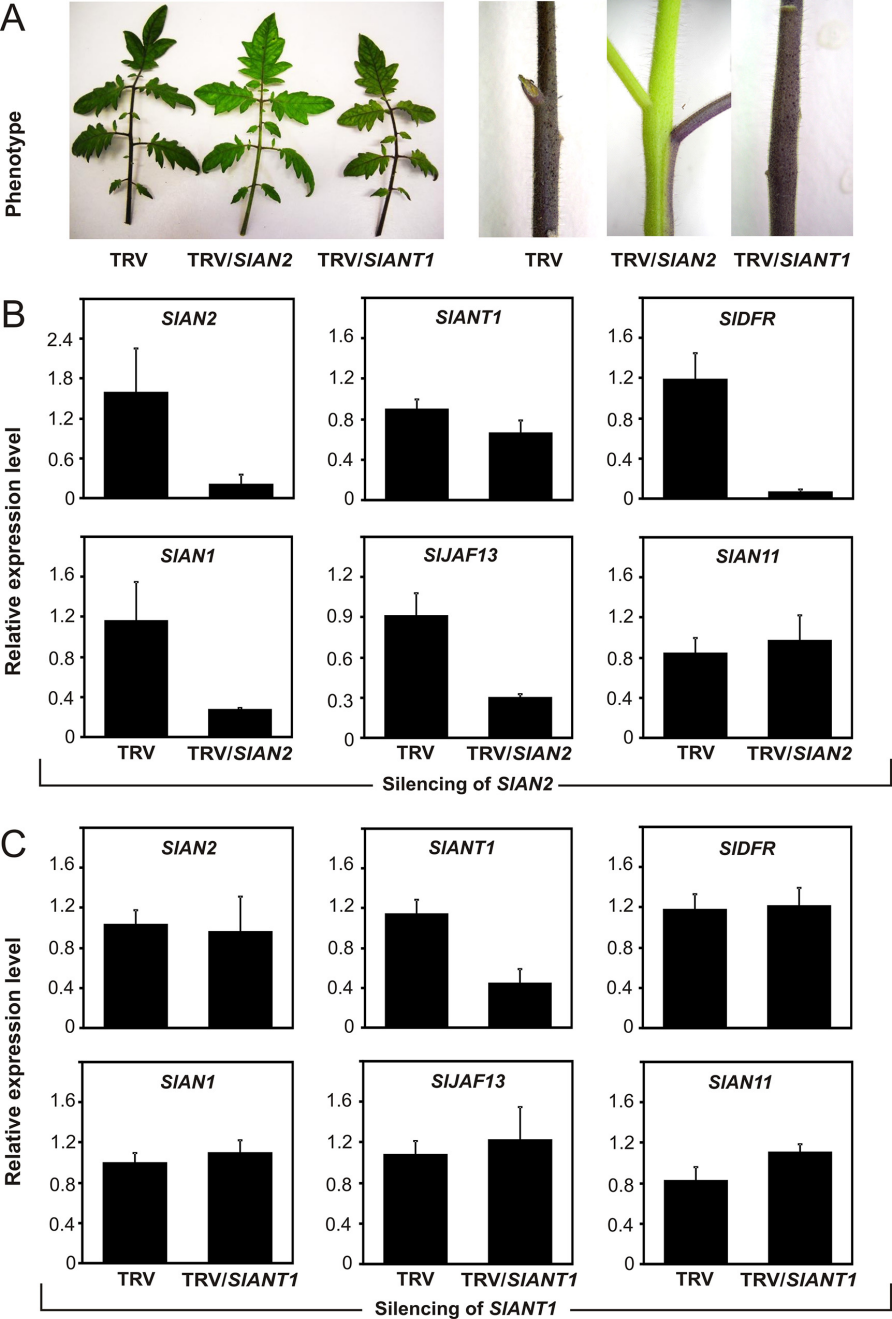
Virus Induced Gene Silencing of *SlAN2* and *SlANT1* in vegetative tissues of tomato plants. Phenotype of *SlAN2* and *SlANT1* silenced leaves and stems (TRV/*SlAN2*, TRV/*SlANT1*, respectively) compared to non-silenced controls (TRV) (A). Quantitative analysis of transcript levels for *SlAN2*, *SlAN1*, *SlAN11*, *SlDFR*, *SlANT1* and *SlJAF13* in *SlAN2* silenced tomato plants (TRV/*SlAN2*) (B) and in *SlANT1* silenced tomato plants (TRV/SlANT1) (C) compared to non-silenced controls (TRV). Expression levels are shown as relative units, with the value of one of the biological replicates of control TRV samples set to one. Data are means of three biological replicates ± SD.

To further elucidate the respective contribution of each of the two MYB genes in the activation of the anthocyanin pathway, tomato seedlings were grown under stress conditions (low temperature plus limiting soil) to promote strong anthocyanin accumulation and Virus Induced Gene Silencing (VIGS) of *SlAN2* or *SlANT1* was carried out. As shown in Fig 5A, the silencing of *SlAN2* caused a strong reduction of anthocyanin accumulation both in leaves and in the stem, as compared to control plants. qPCR analysis showed a strong down-regulation of *SlAN2* mRNA in the silenced tissues, confirming the effectiveness of the VIGS, as well as a strong repression of the expression of *SlDFR* (Fig 5B). Furthermore, silenced *SlAN2* tissues showed significant reduction of the bHLHs *SlAN1* and *SlJAF13* transcripts providing evidence that SlAN2 is involved in the transcriptional regulation of these genes. Interestingly, the expression in petals of the petunia bHLH factor PhAN1 is similarly down-regulated in mutants for the MYB protein PhAN4 [6]. On the contrary, the expression of *SlAN11* was not altered in silenced *SlAN2* tissues, further confirming that *SlAN11* is expressed independently from SlAN2. *SlANT1* was not significantly affected by the silencing of *SlAN2* (Fig 5B), suggesting that this gene did not play an important role in the anthocyanin accumulation observed in the not silenced plants. This was further confirmed by the silencing of *SlANT1* itself. In tomato seedlings growing in the same stressing conditions and accumulating elevated quantities of anthocyanins, the reduced expression of *SlANT1* obtained by VIGS and confirmed by qPCR did not result in loss of pigmentation (Fig 5A) or in changes in the expression of the genes analyzed (Fig 5C). All these results, together with those shown in Fig 4, suggest that SlAN2, together with SlAN1 and probably SlJAF13, is involved in the regulation of anthocyanin biosynthesis in vegetative tissues of tomato plants upon light and cold induction and that *SlDFR* is a target gene of these regulators. Furthermore, the role of SlANT1 in the regulation of pigment accumulation seems to be at most marginal.

## Discussion

In recent years, several attempts were made to increase the nutritional value of tomato fruits by inducing the synthesis of anthocyanins. These flavonoids are indeed normally absent in tomato fruits, although some lines, like *Aft*, produce small amounts of anthocyanins in the fruit skin [25]. Another line, *atv* is instead characterized by high anthocyanin levels in vegetative tissues [25]. The cross between *Aft* and *atv* results in much higher anthocyanin levels in the fruit [30,31]. The product of the *atv* locus is presently unknown, while *Aft* probably encodes a MYB gene, either *SlANT1* [36] or *SlAN2* [34,35]. Attempts to increase the anthocyanin content in tomato fruits were made by expressing anthocyanin regulatory genes from different plants species. The most successful case are the lines obtained by [28] by expressing two snapdragon genes, *Del*, a bHLH-type TF, and *Ros1*, an R2R3 MYB-type TF, obtaining tomato fruits characterized by an intense purple coloration both in the peel and flesh. Recently, two different tomato R2R3 MYB-type TFs involved in anthocyanin synthesis have been identified and their ectopic expression resulted in increased anthocyanin pigmentation in tomato plants [32–36], as confirmed by the results presented here (Fig 2).

In this work we analyzed the role of SlAN2 and SlANT1 within one experimental setting and compared their roles in the induction of pigmentation in different plant parts under growth conditions that result in anthocyanin accumulation. Our results indicate that the SlAN2 protein is as efficient as SlANT1 in inducing anthocyanin synthesis in tomato fruits when their expression is driven by the 35S promoter. Both 35S:*SlANT1* and 35S:*SlAN2* plants displayed high expression of *SlDFR*, encoding a key enzyme of the anthocyanin biosynthetic pathway. This is confirmed by the transactivation of the *SlDFR* promoter in Arabidopsis protoplasts by the two tomato MYBs (Fig 3B). The nuclear localization of both R2R3-MYB factors is in line with the possible involvement of these proteins as TFs (Fig 1C). All these results indicate that the two MYB proteins are functionally active and apparently interchangeable. However, the level of expression of *SlANT1* is low in tomato plants and does not get induced upon exposure to stimuli that result, instead, in higher expression of *SlAN2*. This is based on (i) the analysis of Illumina RNA-Seq data (Fig 1D), (ii) qPCR data (Figs 3–5), and (iii) publicly available microarray data (S4 Fig). These observations suggest different roles for the two MYB factors: *SlAN2* is transcriptionally activated by high light or low temperatures, whereas, surprisingly, *SlANT1* does not respond to these stimuli excluding its involvement in the strong accumulation of pigment under these growth conditions. Silencing of *SlAN2* by VIGS results in reduced anthocyanin biosynthesis and transcription of *SlDFR*, *SlAN1* and *SlJAF13*, whereas no changes were observed following silencing of *SlANT1* (Fig 5). Overall, our results indicate that *SlAN2* induces anthocyanin accumulation in tomato in response to high light and low temperature through the control of the expression of *SlAN1* and *SlJAF13* (Fig 5B). Expression of the WDR gene *SlAN11* seems instead to not require *SlAN2* (Fig 5B), implying that its possible role in the regulation of anthocyanin synthesis is independent from light and temperature. In other plant species the WDR factors involved in anthocyanin synthesis are constitutionally expressed in all plant parts [8] and contribute to the activation/stabilization of the transcription complex by interacting with it [63].

A role of SlANT1 in the synthesis of anthocyanins in tomato is so far not proven. The homology shared by the SlANT1 protein with other MYB regulators of the anthocyanin pathway, the strong induction of anthocyanin accumulation by this protein when ectopically expressed in transgenics (Fig 2), the presence in its promoter region of several LREs and other regulatory elements compatible with anthocyanin synthesis (S6 Fig), and its ability to activate the *SlDFR* promoter in transient assays in Arabidopsis protoplasts (Fig 3B) seem however to indicate that SlANT1 is able to participate to the MBW complex and activate the same target genes induced by SlAN2. It was shown that *SlANT1* expression is responsive to nitrogen depletion [64], while a survey using Genevestigator [65] of 194 different perturbations by microarray analysis reveals that changes in expression of *SlANT1* do not exceed +1.28 fold/-1.2 fold, while expression of *SlDFR* displays changes of +498 fold/-7.7 fold under the same experimental conditions. This suggests that *SlANT1* is poorly responding to environmental clues.

During the evolution of the tomato clade (*Solanum* genus, section *Lycopersicon)* [66] at least 13 tomato species have evolved and occupied various habitats of the western coast of South America, from central Ecuador to northern Chile, including the Galapagos Islands [67]. Only two of them accumulate lycopene in the fruits (*S*. *lycopersicum* and *S*. *pimpinellifolium*), other two produce yellow to orange fruits (*S*. *galapagense* and *S*. *cheesmaniae*), while the others (*S*. *arcanum*, *S*. *chilense*, *S*. *corneliomulleri*, *S*. *pennellii*, *S*. *peruvianum*, *S*. *huaylasense*, *S*. *chmielewskii*, *S*. *habrochaites and S*. *neorickii*) produce green mature fruits which in some cases accumulate anthocyanin pigments to various degrees (S7 Fig). Moreover, as cultivated tomato is a domesticated species, some characters could have gone through very strong selection operated by growers and this is very likely to have involved characters like pigmentation patterns. It is therefore not excluded that the *SlANT1* allele of tomato is a “relique” allele which acquired its low expression level and/or insensitivity to environmental stimuli during speciation/domestication processes. In a previous paper [68], nucleotide and amino acid polymorphisms in *ANT1* gene were detected between AC and the *Aft* genotype, derived from *S*. *chilense*. These polymorphisms were not accompanied by differences in *ANT1* transcription rate, at least in the fruit peel [68]. However, the same authors could not exclude the existence of differences in the promoter regions of *Aft* and *S*. *lycopersicum ANT1* genes leading them to respond differently to the same environmental factor [68]. Furthermore, we used the coding sequences of *SlAN2* and *SlANT1* to identify their orthologs in the *S*. *pennellii* genome database available on SGN [69,40]. As in the case of the *Aft-AN2* allele [54], that derives from *S*. *chilense*, the ortholog of *SlAN2* in *S*. *pennelii*, *SpAN2* (annotated as Sopen10g035640) is conserved (S8 Fig). To the contrary, there is no annotation for the ortholog of *SlANT1* in *S*. *pennellii* (*SpANT1*), since point mutations generate a premature stop codon in the sequence with respect to *SlANT1* and *Aft*-*ANT1* [54,68] (S9 Fig). These data suggest that a low selective pressure acted on *ANT1* during speciation/domestication processes. However only a systematic characterization of these genes in all the wild tomato species would help to elucidate this aspect. Nevertheless, a role for SlANT1 MYB factor in the activation of pigmentation in domesticated tomato under other conditions than those studied here, in different tissues or at different developmental stages of the plant cannot be excluded at this stage.

SlAN2 is therefore the main MYB regulator of anthocyanin biosynthesis in tomato plants in response to stimuli like light and cold.

## Supporting Information

S1 Fig Evolutionary relationships of MYB proteins. The evolutionary history was inferred using the Neighbor-Joining method [42]. The optimal tree with the sum of branch length = 30.36267216 is shown. The percentage of replicate trees in which the associated taxa clustered together in the bootstrap test (1000 replicates) are shown next to the branches [57]. Branches corresponding to partitions reproduced in less than 50% bootstrap replicates are collapsed. The evolutionary distances were computed using the p-distance method [58] and are in the units of the number of amino acid differences per site. The analysis involved 116 amino acid sequences. All ambiguous positions were removed for each sequence pair. There were a total of 786 positions in the final dataset. Evolutionary analyses were conducted in MEGA6 [41].
(TIF)

S2 Fig Evolutionary relationships of bHLH proteins. The evolutionary history was inferred using the Neighbor-Joining method [42]. The optimal tree with the sum of branch length = 37.06676035 is shown. The percentage of replicate trees in which the associated taxa clustered together in the bootstrap test (1000 replicates) are shown next to the branches [57]. Branches corresponding to partitions reproduced in less than 50% bootstrap replicates are collapsed. The evolutionary distances were computed using the p-distance method [58] and are in the units of the number of amino acid differences per site. The analysis involved 112 amino acid sequences. All ambiguous positions were removed for each sequence pair. There were a total of 1083 positions in the final dataset. Evolutionary analyses were conducted in MEGA6 [41].
(TIF)

S3 Fig Evolutionary relationships of WDR proteins. The evolutionary history was inferred using the Neighbor-Joining method [42]. The optimal tree with the sum of branch length = 21.14733530 is shown. The percentage of replicate trees in which the associated taxa clustered together in the bootstrap test (1000 replicates) are shown next to the branches [57]. Branches corresponding to partitions reproduced in less than 50% bootstrap replicates are collapsed. The evolutionary distances were computed using the p-distance method [58] and are in the units of the number of amino acid differences per site. The analysis involved 78 amino acid sequences. All ambiguous positions were removed for each sequence pair. There were a total of 2873 positions in the final dataset. Evolutionary analyses were conducted in MEGA6 [41].
(TIF)

S4 Fig Genevestigator dataset of *SlANT1*, *SlJAF13* and *SlAN11* expression. Expression of *SlANT1* (*Solyc10g08260*.*1*, yellow dots), *SlJAF13* (*Solyc08g0811420*.*2*, two probe sets represented by blue and green dots) and *SlAN11* (*Solyc03g097340*.*1*, red dots) in 6 developmental stages and 15 anatomical parts of tomato plants. A large dataset of microarray analyses was selected and queried using Genevestigator.
(TIF)

S5 Fig Analysis of *SlAN2* promoter. 2 kb nucleotide genomic sequence of *SlAN2* promoter (A). Light, abscisic acid, defense and stress and methyl jasmonate responsive elements are highlighted with different colors. Legend of the different responsive elements (B). The analysis was carried out with the PlantCARE Software. Only a sub-set of the cis-acting responsive elements identified was reported.
(TIF)

S6 Fig Analysis of *SlANT1* promoter. 2 kb nucleotide genomic sequence of *SlANT1* promoter (A). Light, abscisic acid, defense and stress, gibberellin, drought and methyl jasmonate responsive elements are highlighted with different colors. Legend of the different responsive elements (B). The analysis was carried out with the PlantCARE Software. Only a sub-set of the cis-acting responsive elements identified was reported.
(TIF)

S7 Fig Green mature fruits from wild tomato species. The pictures are available on the TGRC website (http://tgrc.ucdavis.edu/index.aspx). A: *S*. *arcanum*, accession LA2813 (photo by Scott Peacock); B: *S*. *chilense*, accession LA2965 (photo by Scott Peacock); C: *S*. *corneliomulleri*, accession LA3157 (photo by Scott Peacock); D: *S*. *pennellii*, accession LA1656 (photo by Rick, Charles M.); E: *S*. *peruvianum*, accession LA2958 (photo by Scott Peacock); F: *S*. *huaylasense*, accession LA1981 (photo by Rick, Charles M.); G: *S*. *chmielewskii*, accession LA3663 (photo by Scott Peacock); H: *S*. *habrochaites*, accession LA1986 (photo by Scott Peacock); I: *S*. *neorickii*, accession. LA2190 (photo by Rick, Charles M.).
(TIF)

S8 Fig Clustal W alignment of the coding sequences of *SlAN2*, *SpAN2* and *Aft-AN2*. Red shading indicates identical sequences.
(TIF)

S9 Fig Clustal W alignment of the coding sequences of *SlANT1*, *SpANT1* and *Aft-ANT1*. The red box indicates the mutated codon that produce a premature stop in *SpANT1*. Red shading indicates identical sequences.
(TIF)

S1 Table Primers used for quantitative RT-PCR analysis. (DOCX)

S2 Table Expression pattern of tomato MYB, bHLH and WDR genes in plants grown in standard conditions. Normalized expression (FPKM) of *SlAN2*, *SlANT1*, *SlAN1*, *SlAN11*, *SlJAF13* and *SlDFR* in different tissues of tomato, analyzed by Illumina RNA-Seq. MG: Mature Green fruit; B: Breaker fruit; B+10: ripe fruit 10 days after breaker stage. Data are the average of two independent biological replicates.
(DOCX)
