# Supplementary figures and images for: Tomato R2R3-MYB Proteins SlANT1 and SlAN2: Same Protein Activity, Different Roles
Source: PLoS One. 2015 Aug 26;10(8):e0136365. doi: 10.1371/journal.pone.0136365 (PMC4556288; doi:10.1371/journal.pone.0136365)

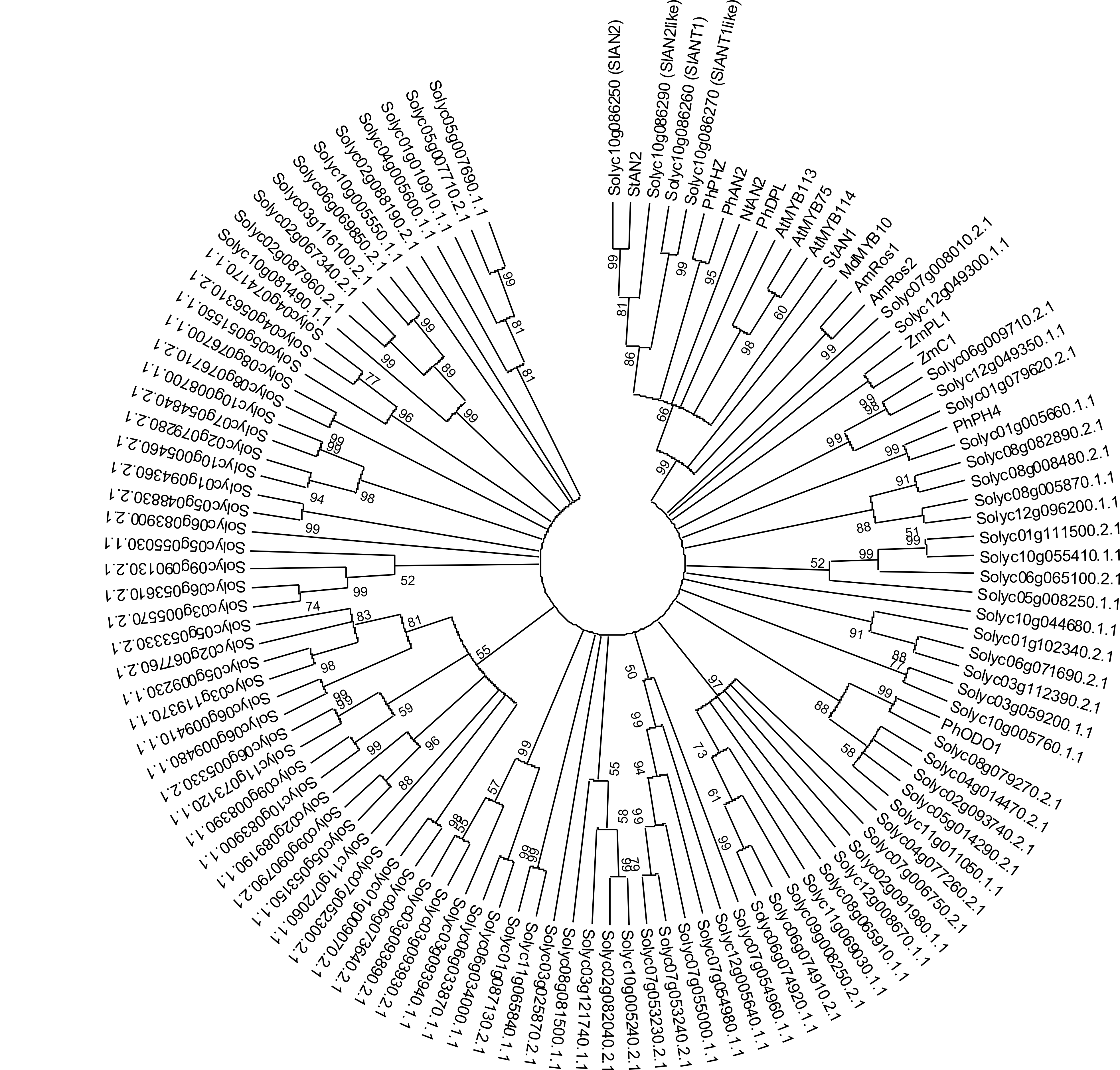

Supplement: S1 Fig — The evolutionary history was inferred using the Neighbor-Joining method [42]. The optimal tree with the sum of branch length = 30.36267216 is shown. The percentage of replicate trees in which the associated taxa clustered together in the bootstrap test (1000 replicates) are shown next to the branches [57]. Branches corresponding to partitions reproduced in less than 50% bootstrap replicates are collapsed. The evolutionary distances were computed using the p-distance method [58] and are in the units of the number of amino acid differences per site. The analysis involved 116 amino acid sequences. All ambiguous positions were removed for each sequence pair. There were a total of 786 positions in the final dataset. Evolutionary analyses were conducted in MEGA6 [41]. (TIF) [file pone.0136365.s001.tif]

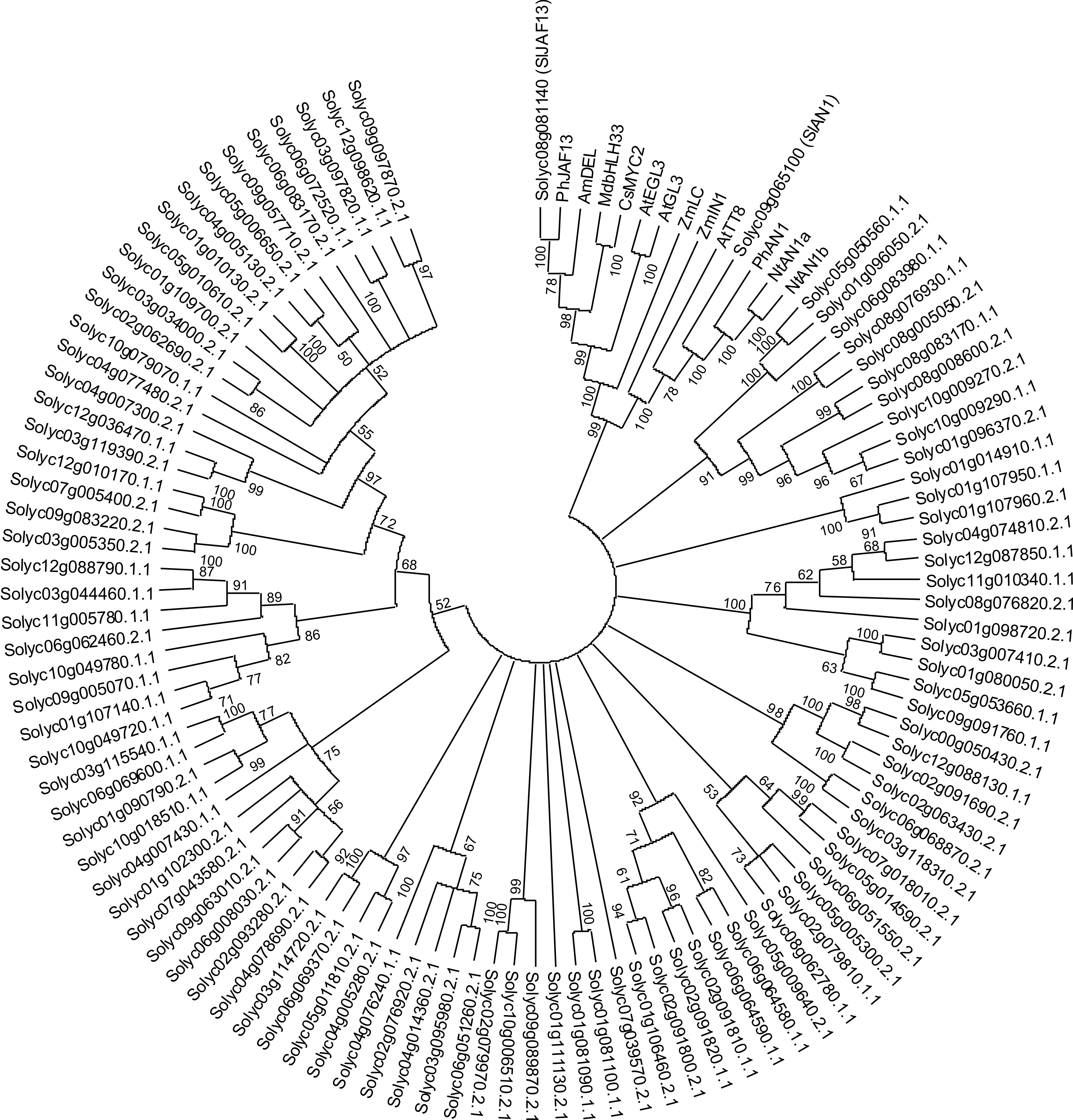

Supplement: S2 Fig — The evolutionary history was inferred using the Neighbor-Joining method [42]. The optimal tree with the sum of branch length = 37.06676035 is shown. The percentage of replicate trees in which the associated taxa clustered together in the bootstrap test (1000 replicates) are shown next to the branches [57]. Branches corresponding to partitions reproduced in less than 50% bootstrap replicates are collapsed. The evolutionary distances were computed using the p-distance method [58] and are in the units of the number of amino acid differences per site. The analysis involved 112 amino acid sequences. All ambiguous positions were removed for each sequence pair. There were a total of 1083 positions in the final dataset. Evolutionary analyses were conducted in MEGA6 [41]. (TIF) [file pone.0136365.s002.tif]

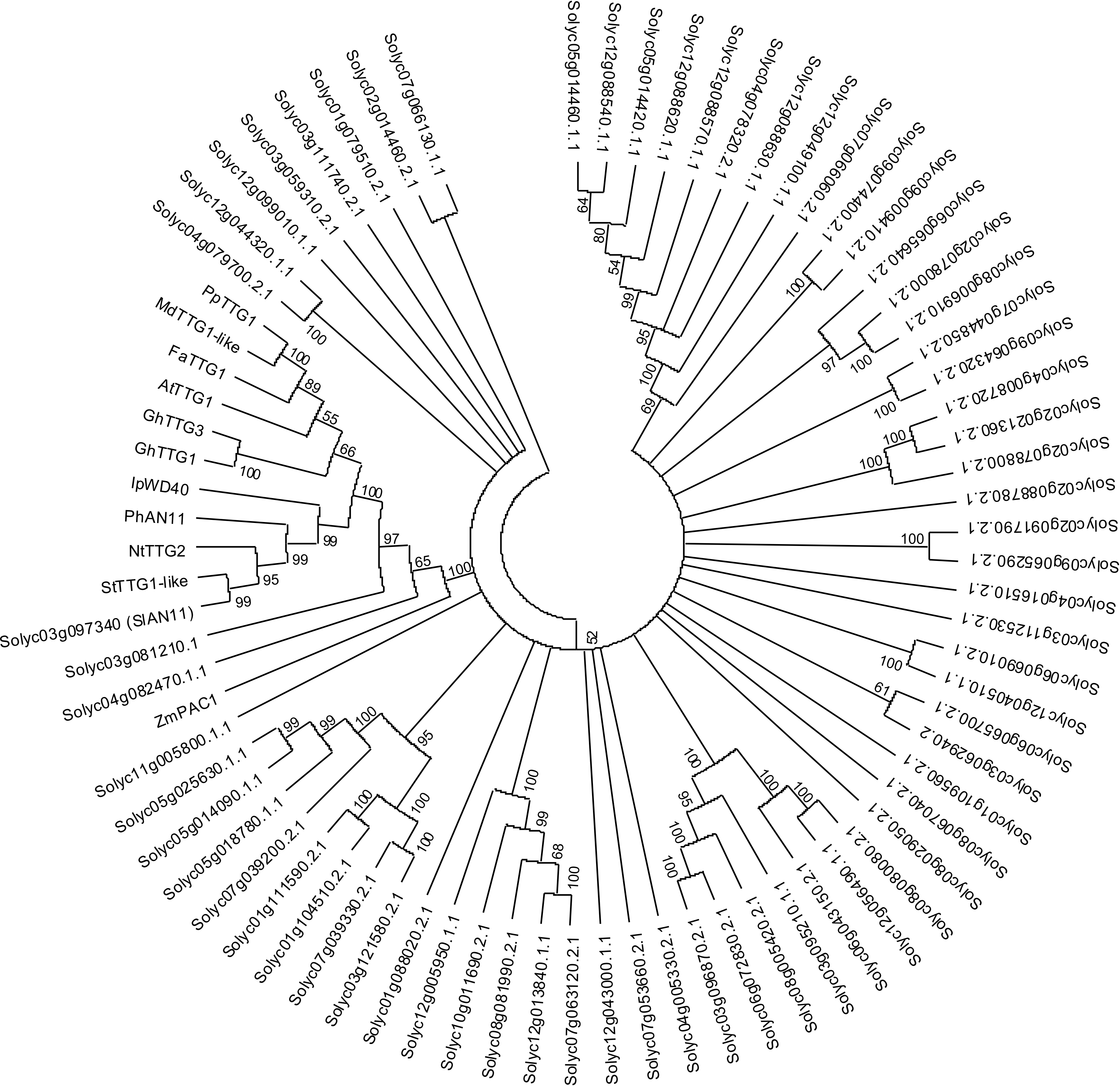

Supplement: S3 Fig — The evolutionary history was inferred using the Neighbor-Joining method [42]. The optimal tree with the sum of branch length = 21.14733530 is shown. The percentage of replicate trees in which the associated taxa clustered together in the bootstrap test (1000 replicates) are shown next to the branches [57]. Branches corresponding to partitions reproduced in less than 50% bootstrap replicates are collapsed. The evolutionary distances were computed using the p-distance method [58] and are in the units of the number of amino acid differences per site. The analysis involved 78 amino acid sequences. All ambiguous positions were removed for each sequence pair. There were a total of 2873 positions in the final dataset. Evolutionary analyses were conducted in MEGA6 [41]. (TIF) [file pone.0136365.s003.tif]

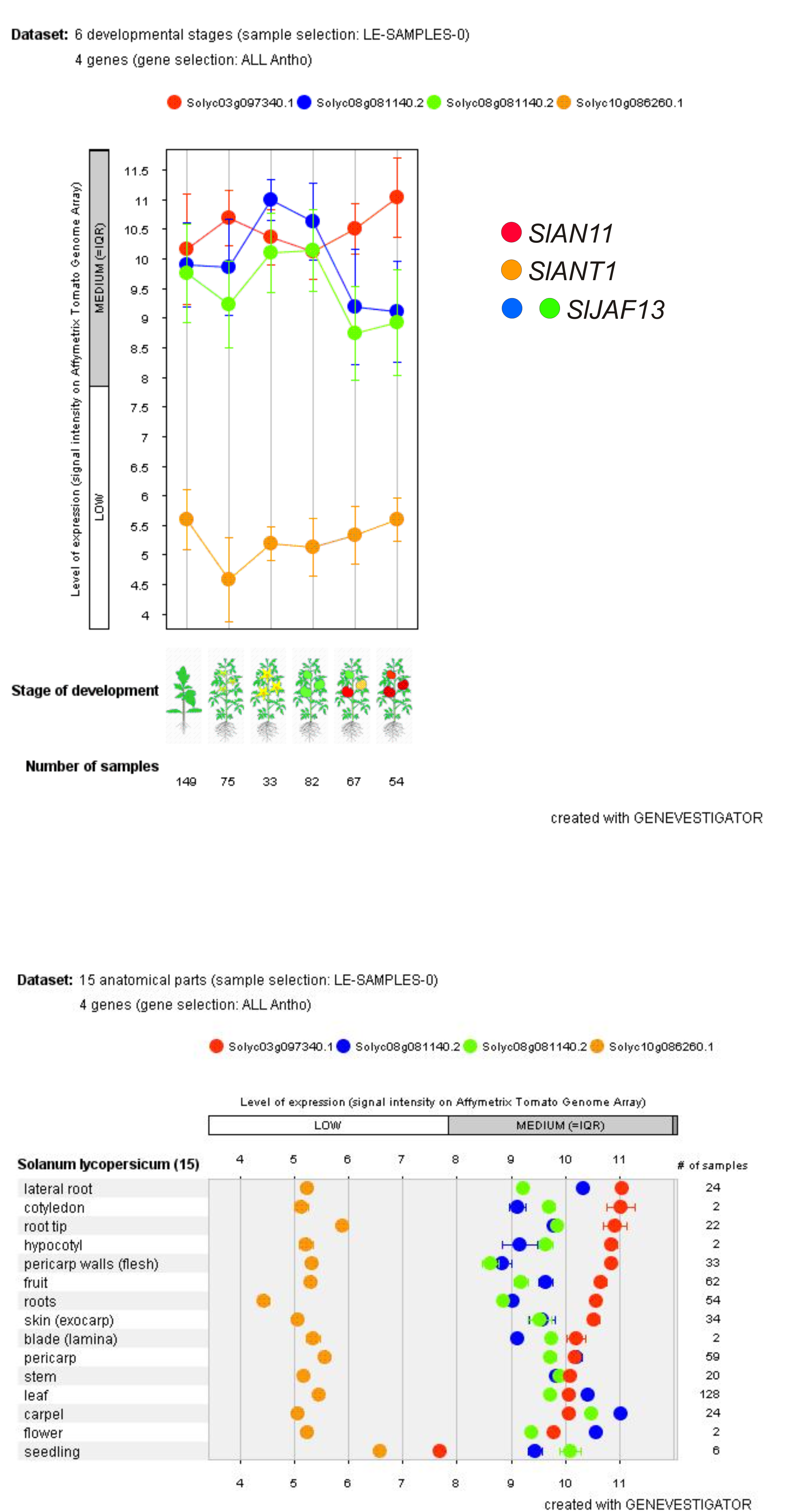

Supplement: S4 Fig — Expression of SlANT1 (Solyc10g08260.1, yellow dots), SlJAF13 (Solyc08g0811420.2, two probe sets represented by blue and green dots) and SlAN11 (Solyc03g097340.1, red dots) in 6 developmental stages and 15 anatomical parts of tomato plants. A large dataset of microarray analyses was selected and queried using Genevestigator. (TIF) [file pone.0136365.s004.tif]

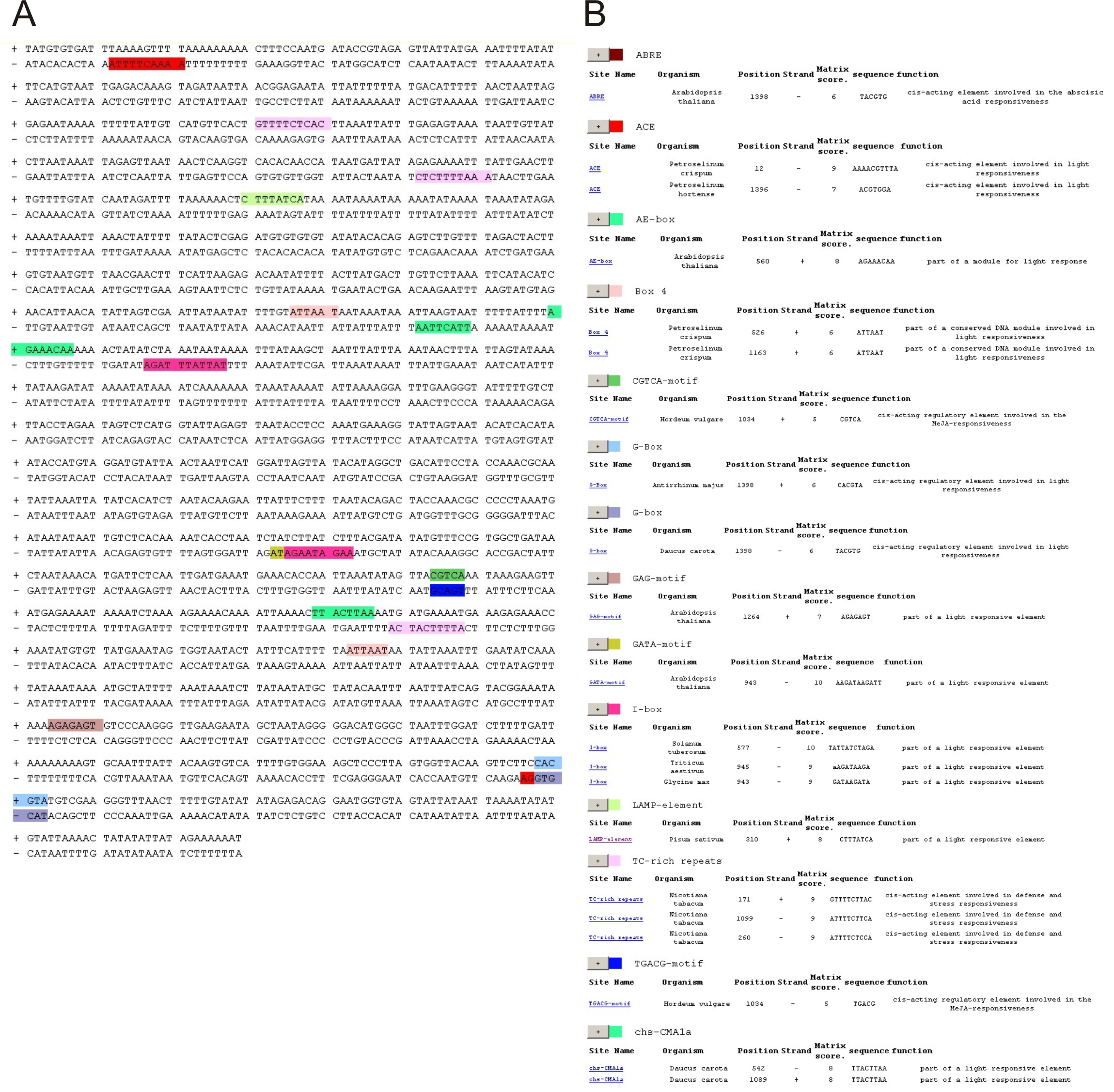

Supplement: S5 Fig — 2 kb nucleotide genomic sequence of SlAN2 promoter (A). Light, abscisic acid, defense and stress and methyl jasmonate responsive elements are highlighted with different colors. Legend of the different responsive elements (B). The analysis was carried out with the PlantCARE Software. Only a sub-set of the cis-acting responsive elements identified was reported. (TIF) [file pone.0136365.s005.tif]

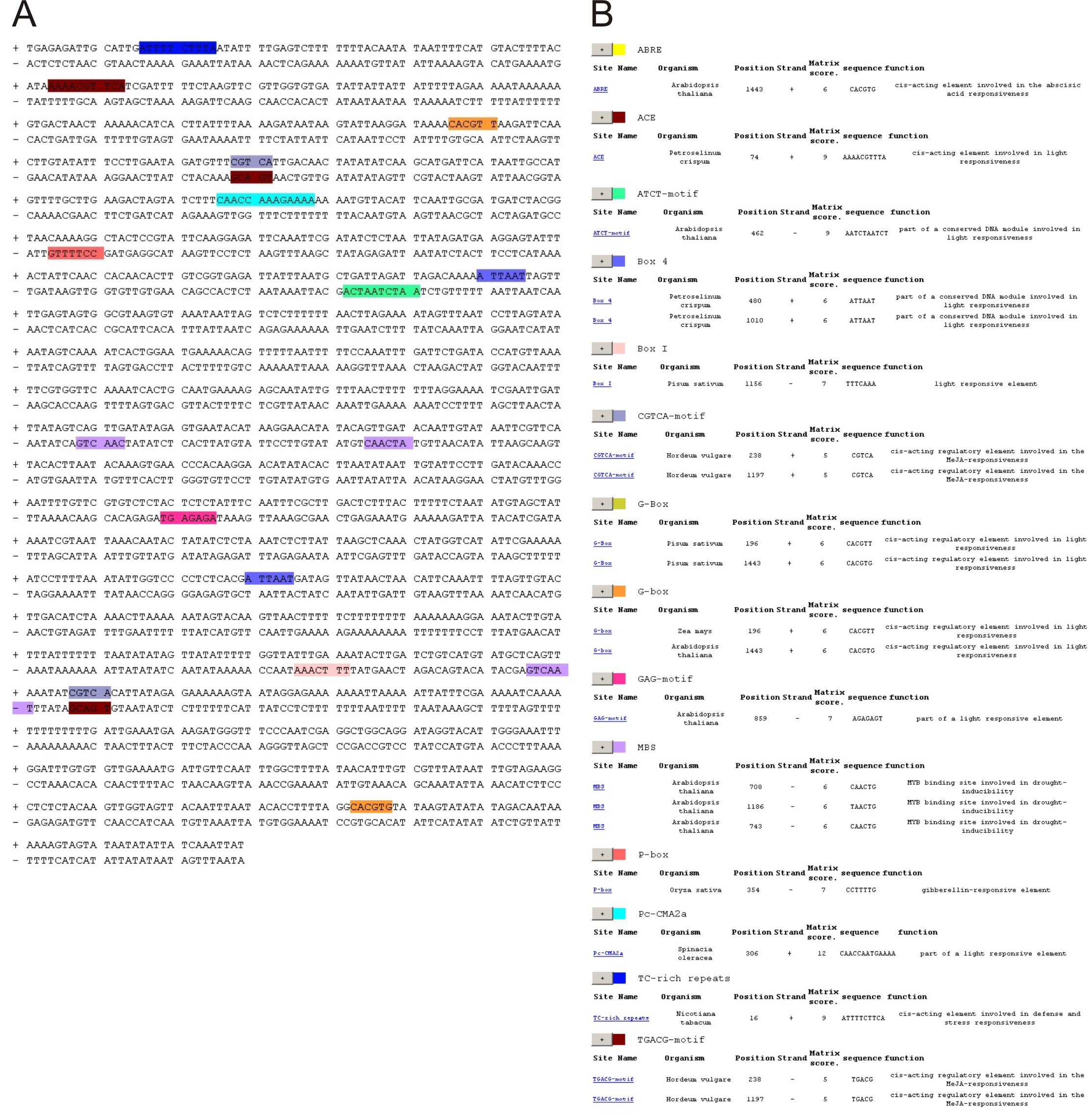

Supplement: S6 Fig — 2 kb nucleotide genomic sequence of SlANT1 promoter (A). Light, abscisic acid, defense and stress, gibberellin, drought and methyl jasmonate responsive elements are highlighted with different colors. Legend of the different responsive elements (B). The analysis was carried out with the PlantCARE Software. Only a sub-set of the cis-acting responsive elements identified was reported. (TIF) [file pone.0136365.s006.tif]

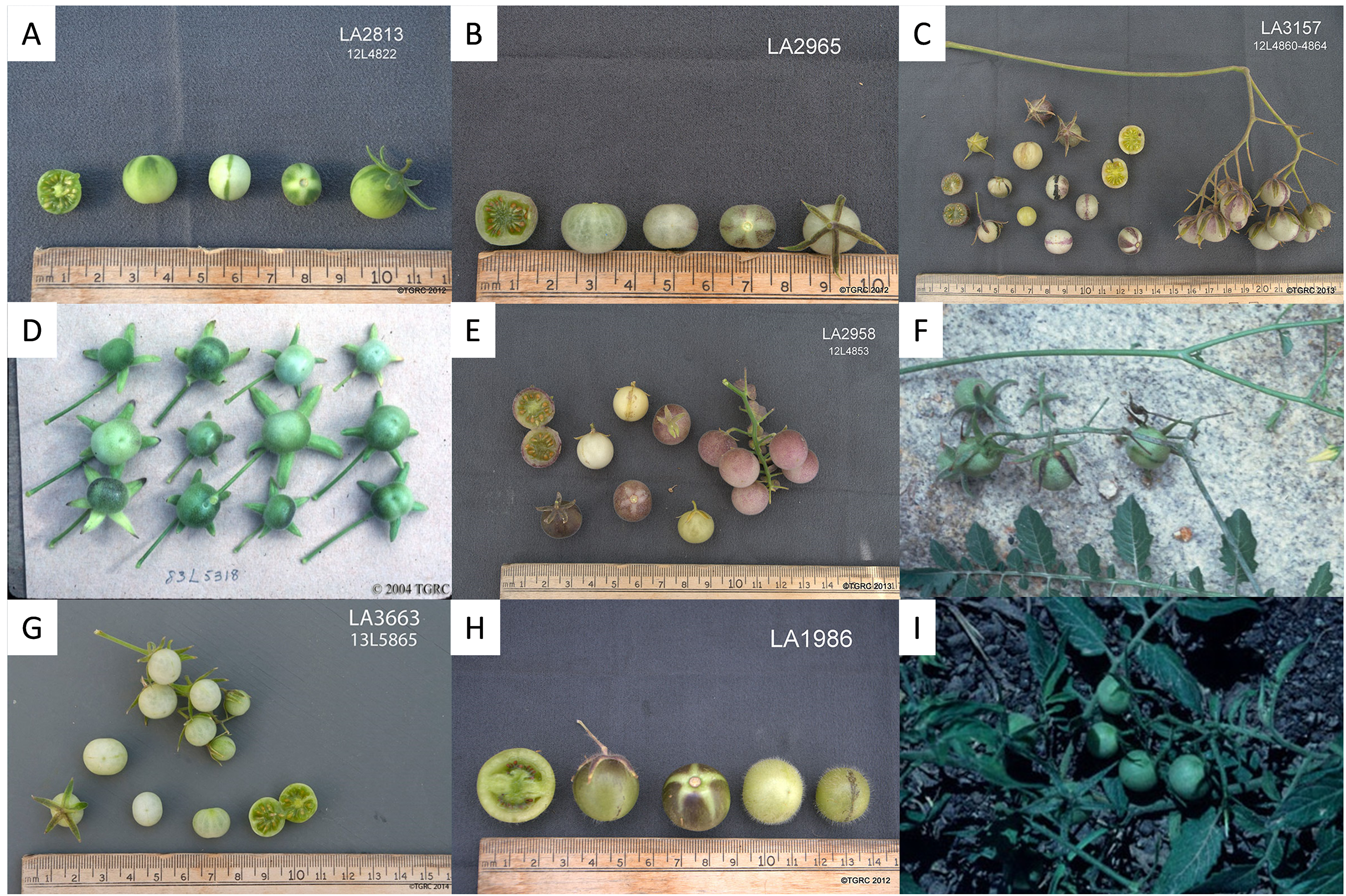

Supplement: S7 Fig — The pictures are available on the TGRC website (http://tgrc.ucdavis.edu/index.aspx). A: S. arcanum, accession LA2813 (photo by Scott Peacock); B: S. chilense, accession LA2965 (photo by Scott Peacock); C: S. corneliomulleri, accession LA3157 (photo by Scott Peacock); D: S. pennellii, accession LA1656 (photo by Rick, Charles M.); E: S. peruvianum, accession LA2958 (photo by Scott Peacock); F: S. huaylasense, accession LA1981 (photo by Rick, Charles M.); G: S. chmielewskii, accession LA3663 (photo by Scott Peacock); H: S. habrochaites, accession LA1986 (photo by Scott Peacock); I: S. neorickii, accession. LA2190 (photo by Rick, Charles M.). (TIF) [file pone.0136365.s007.tif]

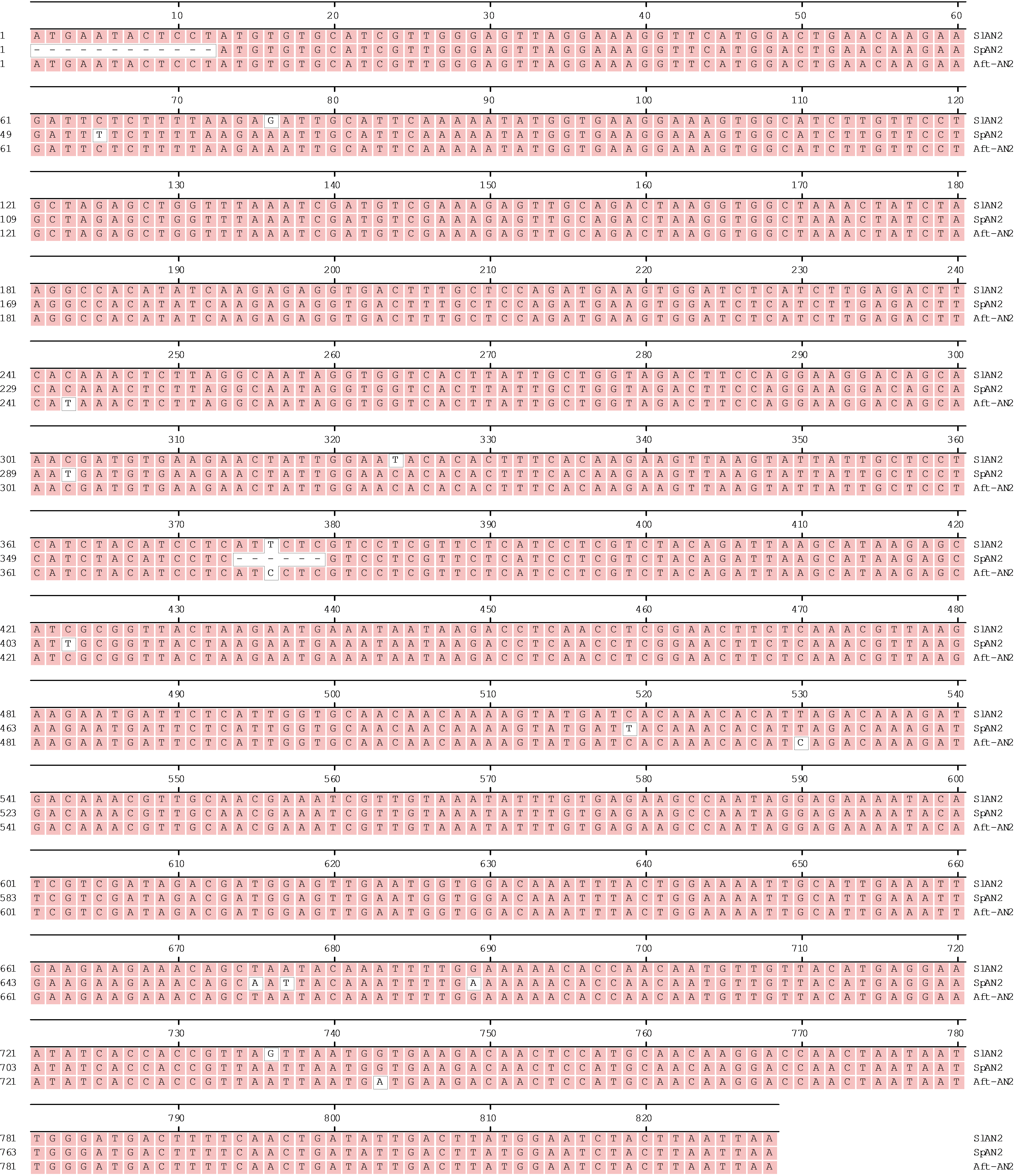

Supplement: S8 Fig — Red shading indicates identical sequences. (TIF) [file pone.0136365.s008.tif]

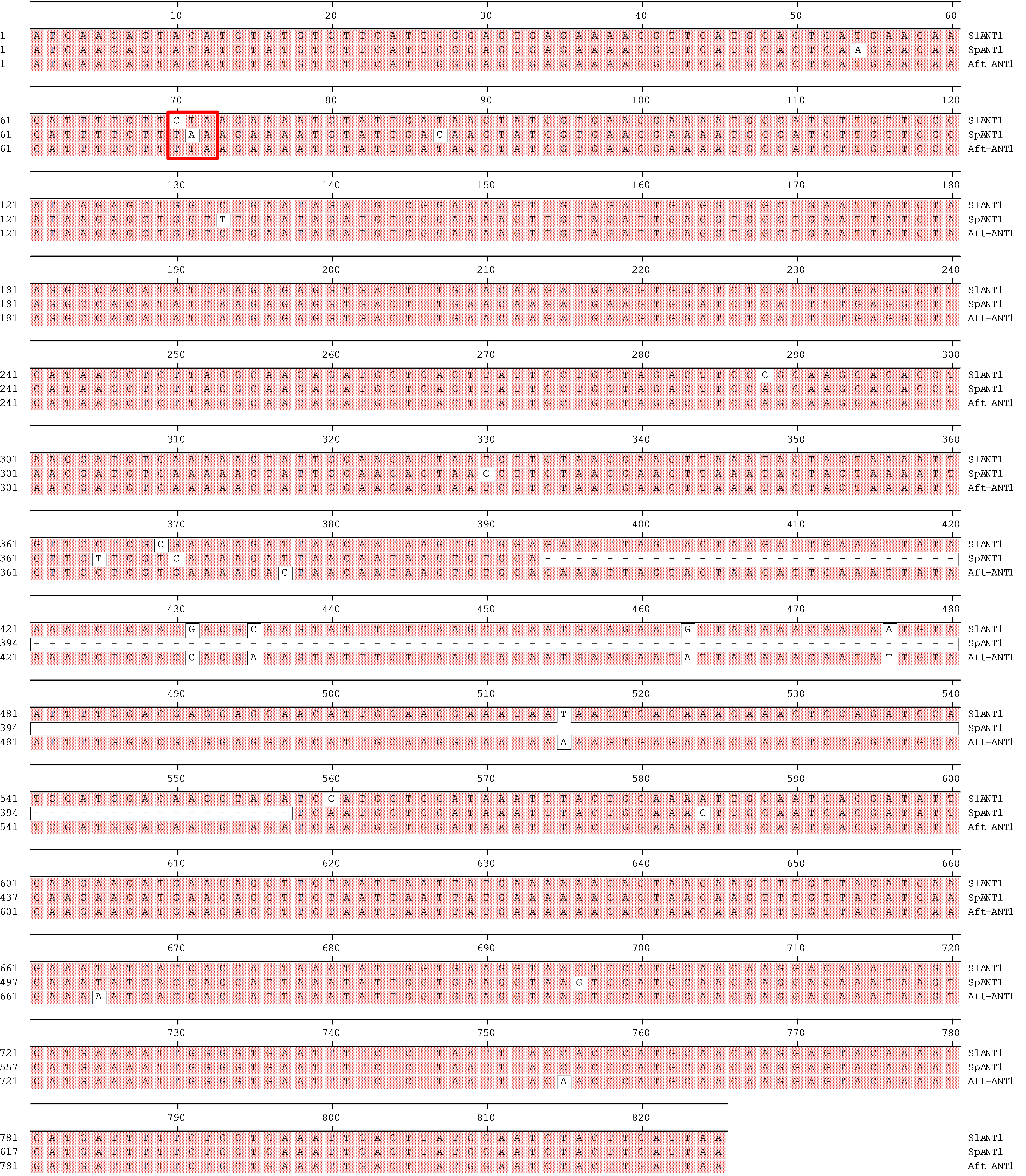

Supplement: S9 Fig — The red box indicates the mutated codon that produce a premature stop in SpANT1. Red shading indicates identical sequences. (TIF) [file pone.0136365.s009.tif]
