# Supplementary material for: Tomato R2R3-MYB Proteins SlANT1 and SlAN2: Same Protein Activity, Different Roles
Source: PLoS One. 2015 Aug 26;10(8):e0136365. doi: 10.1371/journal.pone.0136365 (PMC4556288; doi:10.1371/journal.pone.0136365)
Supplement: S1 Table — (DOCX) [file pone.0136365.s010.docx]

**S1 Table. Primers used for quantitative RT-PCR analysis**

| **Gene Name** | **Forward primer** | **Reverse primer** |
| --- | --- | --- |
| *SlAN2(Solyc10g086250)* | TTCCAGGAAGGACAGCAAAC | AACGAGGACGAGAATGAGGA |
| *SlANT1(Solyc10g086260)* | ATAAGTCATGAAAATTGGGGTGAAT | AGATTCCATAAGTCAATTTCAGCAG |
| *SlAN1(Solyc09g065100)* | CCTCTCTTGGACGGTGTTGT | GCTTGTTGTGGCTCATTGAA |
| *SlAN11(Solyc03g097340)* | ATGAAGTGGAGCCGAGAAGA | TCCATCAGCAGAAACAGA |
| *SlJAF13(Solyc08g081140)* | TCAGGGGATCACTACCGAAC | TCCCATCAAGGTTGGAAGAC |
| *SlDFR(Solyc02g085020*) | TCCGAAGACGACAACGGTTT | TGACAAGCCAAGAGCCGATAA |
| *SlEF1a* (X14449) | GCTGCTGTAACAAGATGGATGC | GGGGATTTTGTCAGGGTTGTAA |
