# Supplementary material for: Tomato R2R3-MYB Proteins SlANT1 and SlAN2: Same Protein Activity, Different Roles
Source: PLoS One. 2015 Aug 26;10(8):e0136365. doi: 10.1371/journal.pone.0136365 (PMC4556288; doi:10.1371/journal.pone.0136365)
Supplement: S2 Table — Normalized expression (FPKM) of SlAN2, SlANT1, SlAN1, SlAN11, SlJAF13 and SlDFR in different tissues of tomato, analyzed by Illumina RNA-Seq. MG: Mature Green fruit; B: Breaker fruit; B+10: ripe fruit 10 days after breaker stage. Data are the average of two independent biological replicates. (DOCX) [file pone.0136365.s011.docx]

**S2 Table. Expression pattern of tomato MYB, bHLH and WDR genes in plants grown in standard conditions**

|  | **root** | **stem** | **leaf** | **flower** | **MG fruit** | **B fruit** | **B+10 fruit** |
| --- | --- | --- | --- | --- | --- | --- | --- |
| *SlAN2*: *Solyc10g086250* | 0.128847 | 3.032550 | 46.700000 | 0.378199 | 0.041626 | 0.000000 | 0.106416 |
| *SlANT1*: *Solyc10g086260* | 0.000000 | 0.000000 | 0.000000 | 0.000000 | 0.000000 | 0.000000 | 0.000000 |
| *SlAN1*: *Solyc09g065100* | 0.022609 | 0.479977 | 0.395981 | 0.051310 | 0.203550 | 0.034146 | 0.000000 |
| *SlAN11*: *Solyc03g097340* | 38.542400 | 22.228000 | 17.501700 | 10.672270 | 23.117300 | 24.962700 | 28.057950 |
| *SlJAF13*: *Solyc08g081140* | 9.120005 | 12.465700 | 15.829700 | 1.931025 | 8.853535 | 5.016015 | 5.348855 |
| *SlDFR*: *Solyc02g085020* | 0.064120 | 4.448245 | 6.430295 | 0.000000 | 0.640215 | 0.000000 | 0.024337 |

Normalized expression (FPKM) of *SlAN2*, *SlANT1*, *SlAN1*, *SlAN11*, *SlJAF13* and *SlDFR* in different tissues of tomato, analyzed by Illumina RNA-Seq. MG: Mature Green fruit; B: Breaker fruit; B+10: ripe fruit 10 days after breaker stage. Data are the average of two independent biological replicates.
